# Production function for modeling hospital activities. The case of Polish county hospitals

**DOI:** 10.1371/journal.pone.0268350

**Published:** 2022-05-12

**Authors:** Agata Sielska, Ewelina Nojszewska

**Affiliations:** Department of Applied Economics, Collegium of Finance and Management, SGH Warsaw School of Economics, Warsaw, Poland; LUMSA: Libera Universita Maria Santissima Assunta, ITALY

## Abstract

The aim of the article is to present the use of production function as a source of knowledge for managers of county hospitals to make rational decisions so as to achieve economic efficiency, including naturally the financial efficiency. The healthcare sector in each country differs from other sectors of the economy. The economically effective operation of county hospitals in Poland is very difficult due to all their determinants. Therefore, all economic analyses should be used to help hospital managers achieve this goal, and production function remains underestimated as a source of knowledge. The Cobb-Douglas and translog production functions were used as sources of knowledge for decision-making by county hospitals. Total number of patient-days was a dependent variable; and the total number of beds, the number of doctors and nurses (in full time equivalents, FTEs) and costs (of materials, electricity, services) were a set of explanatory variables. The significance of explanatory variables most often appeared in models accounting for the workload of nurses. On the other hand, the greatest fit measured with the residual standard error was characterised by models accounting for the number of beds. For each type of production function, the diversified results obtained show the properties of production function. This kind of knowledge is not provided by analyses which are not based on production functions.

## Introduction

### Goals of the research

Hospitals play a significant role in the treatment process and at the same time constitute a group of healthcare providers which take a significant part of health spending [1]. Production function is used to analyse the effectiveness of operation of hospitals, helping in this way to effectively manage hospital’s resources, and thus to properly care for the state of health. The issue of such an application of this function is presented on the basis of the literature, primarily articles, the review of which was based first of all on the PubMed, JSTOR and Springer Link databases.

Hospitals play a special role in the treatment process, i.e. in achieving clinical effectiveness, which translates into the health status of individual patients and the entire society. They implement the most expensive treatment procedures while encountering budgetary constraints, which makes it impossible to meet all health needs and the total demand for health services. Looking from the perspective of striving for clinical effectiveness in the face of shortages of financial resources, material capital and, above all, human capital, striving for the effective use of all these resources is of particular importance. Researching the effectiveness of hospitals is particularly difficult due to the specific nature of their operations. That is why it is so important to use all tools for this purpose. The production function is precisely the tool which—in the opinion of the authors—is used the least frequently. Therefore, in the presented article, attention was focused on demonstrating the possibility of gaining knowledge through the analysis of the production function of hospitals by decision-makers at the hospital and healthcare system level. The aim of the study is to show that the empirical analysis of the production functions of hospitals provides quantitative and qualitative information allowing for making decisions regarding hospital management, its organization and financing. In the current paper we focus on the possibilities presented by the production functions, our goal is not to study the efficiency of the hospitals. The study also analyses which inputs are mostly useful for the modelling of hospital performance.

### Previous studies

The literature review shows that relatively few researchers have used the production functions to analyse the functioning of hospitals and their efficiency. Moreover, the research conducted so far is varied and therefore incomparable, but each point of view brings a new aspect to the knowledge obtained through the analysis of the production function. Therefore, it is worth quoting the results obtained in the previous research.

Authors of some papers focus on the usefulness of using the production function to analyse the functioning of service providers, and above all hospitals. The microeconomic concept of production function links products with the factors employed to produce them. It is used in health economics, and in empirical research this function is estimated with econometric tools. The creator of this analytical approach, which contributes to the understanding of the effectiveness of provision of health services, is Feldstein [2]. According to his approach, a hospital is an enterprise for which costs, productivity and economies of scale can be analysed primarily on the basis of the estimated production function. It is possible and also necessary to get acquainted with the efficient use of resources and production capacity by hospitals. Properly defined hospital products are linked to the factors of production selected for the study using different kinds of functions, but primarily the Cobb-Douglas function [2]. The knowledge of results of this kind of research is of great importance when making decisions with regard, for example, to the allocation of human capital, the training of medical staff (first of all doctors), the size of investment in hospital infrastructure or the distribution of limited hospital capacity among its wards. Using the production function and knowledge of costs, it is possible to determine the different types of costs per unit of product, and this is of particular importance when this kind of unit is a disease case. Thanks to this, it is possible to create benchmarking of hospitals or wards regarding cost effectiveness or quality of treatment in relation to each case mix [2]. Comparing health services on account of the products themselves is not appropriate, because the failure to account for the factors employed to produce them may create a false picture. Thanks to the use of production function for a full analysis of health services, it is also possible to formulate expectations regarding them, deviations from them and from the patterns, which translates into a worse or better quality of services as well as effectiveness of their provision. Owing to the full knowledge gained from production function, indicators can be constructed to measure and evaluate the activities of healthcare providers [3]. An example of such a use of production function can be maternity hospitals due to a certain homogeneity of services, and it is in their case that attempts were made to explain the quality and efficiency of their products with production function. For example, such a study was conducted for 193 English maternity hospitals with the Cobb-Douglas and logarithmic-square functions. It turned out that the most important factors were nurses and the number of beds, although their employment did not achieve technical efficiency, and economies of scale were constant [4].

A large part of the literature is devoted to the possibility of using the production function to study the effectiveness of hospitals. It seems that efficiency is the best concept/tool for operation analysis of enterprises, including healthcare providers which transform factors into products. Production function is a fundamental tool for efficiency analysis; it models the maximum production volume for an employment combination of production factors. It is the function of production that is a critical factor to determine which entrepreneurs are better than others in transforming factors into products [5]. Therefore, some researchers analyse what forms of production function should be used to analyse efficiency, and also formulate new proposals, e.g. flexible form of production function derived from the *Generalized Additive Models* and tested on data from public hospitals in Spanish Galicia during the period 2002–2008 [6–8]. In another example of attempt to improve the analytical tool, which is the form of estimated production function, researchers seek to identify a form which will not only allow to study the inefficiency of hospitals, but also draw economic conclusions through the knowledge of scale elasticity, scale efficiency and which will be serve an effective allocation of resources [9]. The authors’ aim is to provide decision-makers in hospitals with an administrative tool based on the analysis of the stochastic production function, which will enable the identification of non-standard production conditions as well as the determination of a set of corrective actions in the form of proper reallocation of resources. In another study, researchers focused on determining the best form of production function, which the examination of the hospital costs is based on [10]. In their study, they used data from the Washington State hospitals for 1988–1993 to construct long-term cost functions and concluded that the Leontief production function works best, especially when compared to, for example, the translog function. In the next example, attention is focused on the method of determination of the optimal production function to investigate the effect of nurse staffing on patient mortality in acute-care hospitals under Taiwan universal health insurance system [11]. The Random Effect Zero-Inflated Poisson model incorporating a first order autoregressive structure was used for this purpose. The problem of choosing the right analytical tools was also addressed in the next article [12]. The authors focused on creating a new flexible hospital production function using the Generalized Additive Model (GAM) and on the comparison of the results obtained with those provided by the classic Cobb-Douglas model. The databases of public hospitals of the Galician health service for the 2012–2018 time series were used for the calculations made with the use of both tools. The authors of another study used the empirical production function to present the technology used by recipients and living donors on Kidney Exchange platforms to explain the productivity of these platforms due to productivity factors identification [13].

Continuing the topic of efficiency research in the next part of the articles, the production function is a tool for a fuller understanding of its activities by hospitals. In such studies, the problem is the selection of an appropriate functional form. They are usually related to the search for the drivers of ineffectiveness. For example, econometric and linear programming tools are used for this purpose. Production function is a tool for exhaustive analyses, and thus for gaining further knowledge of the performance of such enterprises as hospitals. Particularly interesting results are provided by the model of effects of technical inefficiency, which is one of the most commonly used in the analysis of stochastic frontier function. This is because it provides the ability to estimate technical efficiency specific to each firm, and also combines changes in the functioning of the company with changes in exogenous or conditional variables (e.g. forms of ownership or socio-economic characteristics). In addition, in the panel approach, this model enables the identification of the effects of technical changes and technical efficiency changing over time [14]. Such models of technical inefficiency effects for the stochastic frontier of production function and panel data are used in the analyses of various sectors of the economy and, according to the authors, should also be applied to hospitals [15]. Stochastic frontier analyses are used to study the efficiency of hospitals by using the production as well as cost functions [16]. The use of the cost function also enables a simultaneous estimation of both technical and allocative efficiency, since the cost function is a dual function in relation to production function (under certain conditions) [17]. For example, production function was used in the study of the technical efficiency of private and public hospitals in Turkey [18]. The technical and cost effectiveness of German hospitals was investigated using SFA (Stochastic Frontier Analysis) for production function (Cobb-Douglas) [19]. Many examples could be given of using production function to measure efficiency with SFA. The study of the technical and cost efficiency of Nordic hospitals is an example of using production function through SFA and DEA (Data Envelopment Analysis) [20]. Another example of the use of both of these tools is the study of the effectiveness of government hospitals in Palestine [21]. The calculations revealed that the technical efficiency of these hospitals varied greatly (from 28% to 91%), and in addition, the production process was characterised by declining economies of scale, so the results obtained enable the determination of directions of changes in hospital management; the benchmarkings drawn up should be an incentive to improve the efficiency of Palestinian hospitals. This analysis is particularly important because it concerns the public sector, where the market mechanism does not work and due to it, it is possible to compare best practices in the provision of health services. It is about the improvement of the quality of these services as well as the reduction in their costs down to the necessary minimum—according to the microeconomic definition of the cost function. It is possible thanks to the application of production function, which, thanks to its complexity, contains information about all hospital activities related to health services. This approach is particularly important for university hospitals, which also deal with medical education, clinical research and implementation innovative technologies.

The production function can also be used for cost analysis as shown by some articles. It is worth emphasising that production function may be used to introduce activities to curb the increase in the costs of operations of healthcare providers, primarily hospitals. The exponential increase in these costs, as well as the related forecasts, already in 2010 alarmed the WHO and prompted the encouragement of policymakers and politicians to take action to hinder the adverse trend [22]. In addition, the report shows that up to 40% of healthcare spending is wasted and therefore, it focuses on 10 ways to improve efficiency. Among other things, the emphasis was put on the significance of effective use of all resources in the process of production of health services. All kinds of cost functions are used to analyse hospital costs and attempts to achieve efficiency. The microeconomic cost function shows all the volumes of production made at minimum, i.e. necessary costs. Using a dual approach, it can be concluded that cost functions describe the axial volume of production which is possible to be made at a specific cost level. This position/locus of efficiency is derived from production function as well as from the prices of production factors employed [23]. Thus, cost analyses of hospitals are possible by the isolation of cost function from production function. Cost function can also be used to study efficiency, and therefore some research deals with improving and testing different forms of production functions to obtain the best efficiency results for the cost function derived from them [17].

Many articles [16, 24–27] focus on estimating the productivity of hospitals (thanks to the knowledge of production function) as well as their costs is a fundamental issue in making decisions concerning, for example, how to finance hospitals or introduce appropriate incentives both to reward and punish those who have a positive or negative impact on the hospital performance. In an example of such a study, the aim of the analysis was to find out the determinants of the temporary as well as permanent effectiveness of hospitals [28]. 133 hospitals in Lombardy were examined between 2008 and 2013 to find out that the average total inefficiency was almost 25% higher than shown in the former estimates. This was possible thanks to the distinction between temporary and permanent effectiveness, determination of their meaning and changes.

Investments in IT systems are a prerequisite for the improvement of the quality of health services and therefore their impacts should be diagnosed in detail. The growth rate of spending on it reaches the growth rate of healthcare spending in the US [29, 30]. In the studies devoted to the analysis of this issue, hospital production functions are used, accounting for two types of IT systems: the first of them used in the treatment process and the other in administrative activities [31]. The analysis shows the significance of the implementation itself of IT innovations as well as the ways of implementation; thus, it should all be done in the right order and in the right places. Since a properly implemented information technology (IT) affects the increase in hospital productivity, the next article is devoted to the examination of it and assesses a value-added hospital production function parameters [32]. Endogenous choices of manufacturing factors, i.e. labour, capital, health IT labour, health IT capital were taken into account in order to determine the returns that hospitals obtain from the introduction of health IT. The calculations based on data from almost all hospitals in California from 1997 to 2007 show that thanks to investments in IT, hospitals obtained high marginal products.

The challenge for hospitals is to implement new, lower-value technologies to replace those of greater value, which forces hard budgetary constraints [33]. Out of concern for the quality of services, each country wants to control the implementation of technologies of a lower value, e.g. by imposing cost-effectiveness thresholds. The authors of this article focused on calculating the threshold for Dutch hospitals.

The knowledge gained from the analysis of hospital production function is used to properly manage the resources that the hospital possesses. The approach basing management on the knowledge obtained from production function is pragmatic and serves reduction in the waste of resources [34]. The authors of this article determined a short- and long-term production function in relation to 64 Iranian hospitals in the years 2007–2009 as well as the elasticity of production factors (showing a percentage increase in products due to a 1% increase in the employment of the analysed factors). The results obtained form the basis for the management improvement. Production function was treated as a tool to improve the efficiency of the use of resources in hospitals operating under social security, i.e. it was also about the efficiency of spending public money. This study showed that hospitals should change medical staff management so that this most important factor is employed in an optimal way. The issue of the effectiveness of hospitals in Iran operating under social security was discussed in an article using SFA based on the Cobb-Douglas estimated production function for the years 2008–2015 [35]. The coefficients of elasticity for the selected factors, their marginal products and the marginal rate of technical substitution (MRTS) were also calculated. MRTS between nurses and doctors showed that the burden on hospital costs caused by doctors’ earning can be reduced by increasing the employment of nurses. The calculations obtained show that production in these hospitals is capital-intensive and achieves increasing economies of scale. Inefficiency was reduced to a minimal extent over time, and the knowledge of its causes gave rise to improved efficiency through a properly conducted reform. The next article also estimated the production functions of 67 Iranian public hospitals with the Cobb-Douglas function in order to obtain the knowledge of productivity of employed factors in order to effectively manage them [36]. The flexibility of hospital services in relation to medical staff and beds as well as technical efficiency coefficients were recalculated again. The calculations confirmed the existence of inefficiency, and the knowledge of their carriers should be used to reduce it.

Production function is also used to study the quality of performance of primary health care. In one of the articles, researchers analysed the effectiveness of primary healthcare in Denmark as a result of shortages of GPs within the healthcare system [37]. They tried to identify organisational factors affecting the production of GPs as well as its effectiveness. The calculations showed that it was necessary to introduce thoroughly checked organisational changes, especially with regard to the relationship between nurses and doctors.

The impact of doctors on the production of two types of hospitals, namely those dealing with teaching and those having nothing to do with it, was the subject of another article many years ago [38]. The analysis showed that doctors had a strong and positive impact on the productivity of other factors, and that there was a substitution relationship between them and other resources.

Production function may also be used to study a trade off between the quality of services provided and their number. An example to cite may be an article analysing this problem on the basis of dialysis centres financed by Medicare [39]. Dialyses are increasingly costly procedures, and therefore the knowledge gained from the analysis of the production function accounting for exogenous quality decisions allows for a better solution to the problem of dialysis centres regarding their development and combining a growing number of patients with the safety and quality of dialysis.

Another example of using hospital production function is shown in the article in which the subject of study is the impact of diffusion of technological progress on productivity growth in the treatment of heart attacks [40]. The authors, thanks to hospital production function, built a macroeconomic model to analyse the data of 2.8 million Medicare patients between 1986 and 2004. They proved that hospitals which quickly implement cost-effective innovations achieve significantly better health results of their patients. On the other hand, with a constant, deferred adoption of new technologies, the marginal rate of return on expenses is poor. To sum up, even small differences in the propensity to implement innovative effective technologies lead to a significant diversification in productivity between hospitals.

Production function may also be used to determine the TFP of hospitals [41]. In this way, Japanese hospitals were analysed with panel data from 47 prefectures for hospitals and data from the Secondary Medical Area levels from 1998–2007. The analysis confirmed the existence of economies of scale, as a greater productivity was recorded in larger hospitals.

The analysis of production function may also be used to gain knowledge and opportunities to improve the quality of hospital administration activities [42]. The authors focused on the importance of the stochastic factors they selected, i.e. the transfer of patients between wards caused by the diversity of diseases, the time of reaching the appropriate hospital unit, the number of patients leaving hospital for the efficiency of administration work.

### Contribution

The literature review presented above shows that the range of possibilities of using the production function to analyse the functioning of hospitals is very wide. Current paper contributes to the literature because it is the first study of this type conducted for Polish county hospitals. It shows which inputs contribute to the best fit in such a research and shows estimation results for different production functions. It presents production function as a useful tool for hospital managers and owners interested in gaining knowledge that may allow for making effective decisions regarding hospital organization, management and financing.

## Methods

Since the aim of the article is to present the rationality of the behaviour of producers, such as county hospitals, using the Cobb-Douglas production function and translog function for calculations, it is worth showing what management information can be obtained from these functions.

The analysis made use of the scalar production function [43–46], which is a set of technologically effective processes. It is defined as $f:R_{+}^{2}\to R_{+}^{1}$ (in this simplest terms, two production factors are employed to make the product), where the maximum amount of product is $y\in R_{+}^{1}$ produced by employing a vector of factors of production $x=\left(x_{1},\;x_{2}\right)\in R_{+}^{2}$.

When examining the properties of production function, the characteristics of the production process should be taken into account, i.e. marginal productivity (the pace of production growth and its rate), elasticity, the marginal rate of substitution.

For a producer, an important piece of information is the pace of production growth caused by the change in the employment of *i-th* factor, which is called marginal product (marginal productivity), presented by formula (1).


$$T_{i}\left(x\right)=\frac{\partial f\left(x\right)}{\partial x_{i}}$$ (1)

A producer knows how the volume of production will increase/decrease/remain unchanged with an increase in employment of *i*-th factor by a "unit" (this is an ideal approximation within the frontier, i.e. this "unit" tends to zero), *ceteris paribus*. In microeconomics, the assumption of producer’s rationality implies non-negativity of the marginal product. The producer will not increase the input of the factors of production (which is associated with an increase in costs) if this causes a decrease in the volume of production. Acting in this way would be ineffective. Additional information is provided by the production growth rate in relation to *i*-th factor, defined by formula (2).


$$S_{i}\left(x\right)=\;\frac{\partial f\left(x_{1},\;x_{2}\right)}{\partial x_{i}}\frac{1}{f\left(x\right)}=\frac{T_{i}\left(x\right)}{f\left(x\right)}$$ (2)

Being aware of it, the producer knows by how many percent the production will increase/decrease or remain unchanged with an increase in employment of *i*-th factor by a "unit", *ceteris paribus*. Another tool for the entrepreneur is the elasticity of production in relation to the increase in employment of *i*-th factor, which is presented by the expression (3).


$$E_{i}\left(x\right)=\frac{\partial f\left(x\right)}{\partial x_{i}}\frac{x_{i}}{f\left(x\right)}=S_{i}\left(x\right)x_{i}$$ (3)

This elasticity indicates by how many percent the production will increase/decrease or remain unchanged with an increase in employment of *i*-th factor by 1%, *ceteri*s *paribus*. The expression shows that this elasticity is a dimensionless marginal productivity.

The elasticity of production with respect to the increase in employment of *i*-th factor can be calculated with the logarithmic derivative (4).


$$E_{i}\left(x\right)=\frac{\partial f\left(x\right)}{\partial x_{i}}\frac{x_{i}}{f\left(x\right)}=T_{i}\left(x\right)\frac{x_{i}}{f\left(x\right)}=\frac{\partial lnf\left(x\right)}{\partial lnx_{i}}$$ (4)

The interpretation does not change. As for the marginal product, in the case of a rational producer who maintains the efficiency of manufacturing processes, all these values are non-negative. A measure called economies of scale is defined as the sum of the above elasticities: $RTS=\overset{m}{\underset{i=1}{\sum }}E_{i}\left(x\right)$, which means that the entrepreneur derives additional knowledge from the elasticity of production in relation to the scale of outlays showing by how may percent production will increase/decrease or remain unchanged while increasing the employment of all factors by 1%, as shown in the formula (5).


$$\begin{array}{l} E_{\lambda }\left(x_{1},\;x_{2}\right)=\underset{\Delta \lambda \to 0}{\text{lim}}\frac{\frac{f\left(\left(1+\Delta \lambda \right)x\right)-f\left(x\right)}{f\left(x\right)}}{\frac{\Delta \lambda }{\lambda }}=\;\underset{\lambda \to 1}{\text{lim}}\;\underset{\Delta \lambda \to 0}{\text{lim}}\frac{\frac{f\left(\left(\lambda +\Delta \lambda \right)x\right)-f\left(\lambda x\right)}{f\left(x\right)}}{\frac{\Delta \lambda }{\lambda }}=\\ =\;\underset{\lambda \to 1}{\text{lim}}\;\underset{\Delta \lambda \to 0}{\text{lim}}\frac{f\left(\left(\lambda +\Delta \lambda \right)x\right)-f\left(\lambda x\right)}{\Delta \lambda }\;\frac{\lambda }{f\left(\lambda x\right)}=\underset{\lambda \to 1}{\text{lim}}\frac{\partial f\left(\lambda x\right)}{\Delta \lambda }\frac{\lambda }{f\left(\lambda x\right)} \end{array}$$ (5)

For a producer, it is also important to be able to replace one factor with another, especially with a relative change in their prices, i.e. substitutability. The ratio in which one factor can be replaced by another in the vector of factors: $x=\left(x_{1},\;x_{2}\right)\in R_{+}^{2}$ at the same level of production: *y* = *const* > 0, is indicated by the marginal rate of technical substitution, which the shown in the formula (6), i.e. the quotient of marginal products of employed factors.


$$\sigma_{12}\left(x_{1},x_{2}\right)=\underset{\begin{array}{c} \Delta x_{1}\to 0\\ \Delta x_{1}<0 \end{array}}{\text{lim}}\frac{\Delta x_{2}}{\Delta x_{1}}≅-\frac{dx_{2}}{dx_{1}}$$ (6)

Therefore, the entrepreneur knows how much the employment of the second factor should be increased while reducing the employment of the first factor by a unit, while maintaining the production volume at the current level. In the next step, the entrepreneur should calculate the elasticity of substitution of the first factor by the second factor in the vector of factors: $x=\left(x_{1},\;x_{2}\right)\in R_{+}^{2}$ at the same level of production: *y* = *const* > 0, presented by the formula (7).


$$\varepsilon_{12}\left(x_{1},x_{2}\right)=-\underset{\begin{array}{c} \Delta x_{1}\to 0\\ \Delta x_{1}<0 \end{array}}{\text{lim}}\frac{\frac{\Delta x_{2}}{x_{2}}}{\frac{\Delta x_{1}}{x_{1}}}=-\underset{\begin{array}{c} \Delta x_{1}\to 0\\ \Delta x_{1}<0 \end{array}}{\text{lim}}\frac{\Delta x_{2}}{\Delta x_{1}}\frac{x_{1}}{x_{2}}≅-\frac{dx_{2}}{dx_{1}}\frac{x_{1}}{x_{2}}$$ (7)

This elasticity shows by how many percent the employment of the second factor should be increased, while the employment of the first is reduced by 1% with the volume of production unchanged. It can be seen that the elasticity of substitution is the elasticity of the marginal rate of substitution relative to the quotient of production factors.

In the examination of the production function of county hospitals, two types of production function were used, namely the Cobb-Douglas function (which is a special case of the power function of production with a degree of homogeneity equal to 1) and the translog function.

The Cobb-Douglas function for *m* production factors can be written with an expression (8).


$$y=\alpha_{0}x_{1}^{\alpha_{1}}\ldots x_{m}^{\alpha_{m}}=\alpha_{0}\prod_{i=1}^{m}x_{i}^{\alpha_{i}}$$ (8)

Once logarithmed, it takes the form (9).


$$ln\left(\alpha_{0}\prod_{i=1}^{m}x_{i}^{\alpha_{i}}\right)=ln\alpha_{0}+\sum_{i=1}^{m}\alpha_{i}lnx_{i}$$ (9)

Thus, the elasticity of production in relation to the increase in employment of *i*-th factor is presented by the expression (10), which shows that this elasticity does not depend on the size of the employment of factors.


$$E_{i}\left(x\right)=\frac{\partial \left(ln\alpha_{0}+\sum_{j=1}^{m}\alpha_{j}lnx_{j}\right)}{\partial lnx_{i}}=\alpha_{i}$$ (10)

The marginal product is described by the expression (11), which informs that marginal products depend on the employment of factors of production. If the marginal product is to be positive, it is enough that α_*i*_ > 0. Economies of scale that are the sum of elasticity of production relative to the increase in the employment of factors can be written as follows: $RTS=\prod_{i=1}^{m}\alpha_{i}$, which means that they do not depend on employment of factors.


$$T_{i}\left(x\right)=\alpha_{i}\frac{y}{x_{i}}=\alpha_{i}\frac{\alpha_{0}\prod_{j=1}^{m}x_{j}^{\alpha_{j}}}{x_{i}}$$ (11)

The marginal rate of technical substitution, which is the quotient of the marginal products, is presented by the expression (12), from which it follows that it depends on the size relations of the factors employed.


$$\sigma_{ij}\left(x_{i},x_{j}\right)=\frac{\alpha_{i}}{\alpha_{j}}\frac{x_{j}}{x_{i}}$$ (12)

The elasticity of substitution can be derived as shown in expression (13); thus, the elasticity of substitution does not depend on the employment of factors and equals 1.


$$\varepsilon_{ij}\left(x_{i},x_{j}\right)=\;\frac{\partial \left(x_{j}/x_{i}\right)}{\partial \sigma_{ij}\left(x_{i},x_{j}\right)}\frac{\sigma_{ij}\left(x_{i},x_{j}\right)}{x_{j}/x_{i}}=\frac{\partial \left(\sigma_{ij}\left(x_{i},x_{j}\right)\frac{\alpha_{j}}{\alpha_{i}}\right)\frac{\alpha_{i}}{\alpha_{j}}\;\;\frac{x_{j}}{x_{i}}}{\partial \sigma_{ij}\left(x_{i},x_{j}\right)\frac{x_{j}}{x_{i}}}=\frac{\alpha_{j}}{\alpha_{i}}\frac{\frac{\alpha_{i}}{\alpha_{j}}\;\;\frac{x_{j}}{x_{i}}}{\frac{x_{j}}{x_{i}}}=1$$ (13)

The second functional form used was the translog function, given by the formula (14), often occurring in a logarithmed form, facilitating estimation (15).


$$y=a_{0}x_{1}^{a_{1}}x_{2}^{a_{2}}\ldots x_{m}^{a_{m}}e^{\sum_{i=1}^{m}\sum_{j=1}^{m}b_{i}(log\;x_{i}\;log\;x_{j})}$$ (14)

$$logy=\text{log}\left(a_{0}\right)+a_{1}x_{1}+a_{2}x_{2}+\cdots +a_{m}x_{m}+\sum_{i=1}^{m}\sum_{j=1}^{m}b_{i}(log\;x_{i}\;log\;x_{j})$$ (15)

Marginal products are defined by the expression (16).


$$\frac{\partial y}{\partial x_{k}}=\text{exp}\left(\text{log}\left(\alpha_{0}\right)+\sum_{i=1}^{m}a_{i}\;\text{log}\left(x_{1}\right)\;+\sum_{i=1}^{m}\sum_{j=1}^{m}b_{i}(log\;x_{i}\;log\;x_{j})\right)\frac{1}{x_{k}}\left(\sum_{i=1,i\neq k}^{m}b_{i}\;\text{log}\left(x_{i}\right)+2b_{k}\;\text{log}\left(x_{k}\right)\right)$$ (16)

Each of the 2 functions mentioned was estimated in a form that accounted for two and three independent variables (factors of production). Every time, the dependent variable was the total number of patient-days, while the set of regressors accounted for the total number of beds, the employment of doctors and nurses (both in FTEs) and the costs of materials, electricity and outsourced services. For each model, it is assumed that the set of explanatory variables (consisting of two or three elements) must account for at least one of the following factors: total number of beds, employment of doctors or employment of nurses.

Production functions were estimated with the R version 4.0.2 (2020-06-22)–"Taking Off Again [47] using the least squares method, while robust regression has been done using MASS package [48]. A further analysis was carried out with an MS Excel spreadsheet.

## Data

The data used in the study are from 2018. The sample included 94 county (powiat) hospitals, i.e. hospitals whose founding body is the *powiat* (the second-level unit of local government and administration in Poland). Hospitals come from all over the country. Data used in the current study were provided by the Polish Association of Employers of Powiat Hospitals (OZPSP—Ogólnopolski Związek Pracodawców Szpitali Powiatowych).

The sample used in the analysis includes hospitals of different types. There are both public and non-public hospitals, hospitals which have an Emergency Department or an Intensive Care Unit in their structure as well as those which do not run such units. Functioning of a hospital likely depends on such characteristics, which in turn may lead to the estimates being biased, but the authors decided not to subset the sample due to the following reasons. Firstly, imposing other conditions on the dataset would result in the number of observations decreasing rapidly. When we include only relatively big (i.e. hospitals which have the number of beds between the first and the third quartile) public hospitals with an Emergency Department and an Intensive Care Unit the number of units is below 30. Secondly, our main goal is to show the properties of production function which may prove useful in the analysis of hospitals and their functioning. Aiming for the consistency of this paper, we do not intend to analyse differences between various groups of hospitals which is an interesting and important sphere of future analysis. It is also important for us to analyse, which inputs are mostly useful for modelling. We use robust regression in order to neutralize the effect of potential outliers in the dataset. The values of descriptive statistics for the analysed sample are presented in Table 1. Hospitals in the sample had the number of beds within the range of 57–599, which translates into patient-days in the range of 14.25–154.38. The average employment of doctors and nurses amounted to 121.71 and 383.63 FTEs respectively. On average, the value of materials was 86.389; electricity 10.076, and outsourced services 160.95. It is worth noting that in the case of all variables, the average values were higher than the medians, which indicates the occurrence of right-sided asymmetry and outlaying observations characterised by relatively large values of the analysed variables. Based on the comparison of averages and standard deviations, it can be concluded that the variables were most diverse due to the employment of doctors and nurses and least diverse due to the number of beds.

**Table 1:** Descriptive statistics for the analysed hospitals.

	Total number of patient-days	Total number of beds	materials	electricity	Doctors	Nurses	Outsourced services
	[in thousands]		[100,000 zlotys]	[100,000 zlotys]	FTEs	FTEs	[100,000 zlotys]
min.	14.25	57	8.898	1.617	0.67	56.38	22
mean	60.2	247.9	86.389	10.076	121.71	383.63	160.95
median	53	225.5	59.893	7.395	56	189.47	137.4
max.	154.38	599	406.307	44.762	2325.48	6320.45	457.62
sd	33.1352	119.5885	83.6697	7.8078	267.9383	777.5599	91.0181

Calculated based on the data from Polish Association of Employers of Powiat Hospitals.

In the analysis, we assume the level of significance of α equal to 0.05.

## Results

The results of the estimation of two-factor Cobb-Douglas function are presented in Table A in S1 Appendix. The results show that the model with the costs of electricity and the workload of nurses as explanatory variables can be mentioned as the best description of the operation of hospitals as in this case, both explanatory variables were significant, and the parameter estimates had positive signs. The only other model with two significant inputs has higher residual standard error. It is worth mentioning that some of the models, such as the model accounting for the workload of doctors and nurses as well as the model with the total number of beds and the cost of outsourced services, did not meet the assumptions of production function (negative elasticity, and consequently negative values of marginal productivity). This can be interpreted as inefficiency caused by the employment of such a combination of these factors. In addition to fragments of the production function illustrating the efficient use of production factors, negative marginal products appear, which indicates the need to control the relations of the employed factors in order to avoid hospital operation inefficiency.

The results of the estimation of three-factor Cobb-Douglas function are presented in Table B in S1 Appendix. They show that the best fit assessed by the residual standard error was achieved in the case of a model that accounts for the number of beds, the workload of doctors and the material costs. However, not all variables are significantly different from zero in this case. There are also models which do not meet the assumptions of production function due to the negative elasticity of production in relation to some of the inputs. This means a reduction in the hospital production—the total number of patient-days. Thus, the availability of health services for patients is becoming limited. The results of the estimation of two-factor translog function are presented in Table C in S1 Appendix. Based on the residual standard error value, the model with the number of beds and doctors as explanatory variables can be considered the best description of the operation of hospitals. Unfortunately, only one estimate is statistically significant in this case.

The results of the estimation of three-factor translog function are presented in Table D in S1 Appendix. The best fit evaluated with residual standard error was achieved with a model which accounts for the number of beds, costs of electricity and workload of doctors. Unfortunately, in this model, only two estimates of inputs were statistically significant, and in addition, this model does not meet the assumptions of production function due to the negative marginal productivity of the employment of doctors, and thus also a negative elasticity of the production function.

## Discussion

Table A in S2 Appendix presents additional sources of knowledge resulting from the two-factor Cobb-Douglas function. For all types of models we will provide sample interpretations for the models characterized by the greatest number of significant parameters (providing it satisfies all the conditions for the production function, such as positive productivities). For the two-factor estimations of the Cobb-Douglas function, we will discuss the values of marginal productivity and other functions for the model with electricity and nurses as explanatory variables. According to the principles of economic analysis, in the interpretation, we use the principle of *ceteris paribus*, which means that we abstract from the standards that may be applicable in a specific healthcare system (e.g. the relation of the number of medical staff to the number of beds). It should be emphasised that data from county hospitals also result from the regulations of the Ministry of Health in (e.g. the relation of the number of medical staff to the number of beds). It appears that when comparing the results of health systems in different countries, it should be borne in mind that they are to some extent incomparable. The standards introduced by law create conditions to achieve efficiency, and their diversity between countries is an obstacle to modelling their own organisational, management and financial solutions, because these detailed legal regulations must be followed. This is a necessary simplification in order to illustrate the possibilities offered by the production function for the analysis of the situation in the entity and when making decisions. The results indicate that the electricity marginal productivity for the average and median amounted to 3.0993 and 3.1562 respectively. This means that 1 extra unit of money spent on the electricity would allow for an increase in patient service of about 3 patient-days, with other factors unchanged. The marginal products of FTEs for nurses are equal to 0.0342 and 0.0517 (again for averages and medians respectively),which means that an increase in nurse employment by 1 FTE would allow, with other factors unchanged, for an increase in patient service of about 0.03–0.05 patient-day. Production growth rates mean that the capacity to serve patients calculated in patient-days will increase by around 0.05%-0.06% if the cost of electricity is increased by a unit, and by 0.0005%-0.0011% if the employment of nurses is increased by 1 FTE. The elasticity of production in relation to the increase in the employment of manufacturing factors means that a one-percent increase in the costs of electricity translates into an increase in the capacity to serve patients (in patient-days) by 0.48%, and a one-percent increase in the number of FTEs by 0.2%. Economies of scale for the discussed function do not exceed 1, which means that a 1% increase in outlays in both factors will result in less than 1% more patient service capacity. The marginal substitution rate calculated for average values of 0.011 informs about the need to increase the employment of nurses by nearly 0.011 FTEs in the situation of reducing the electricity costs by 1 unit and the intention to maintain patient service at the current level. In the case of medians, the necessary increase in employment amounts to about 0.016 FTEs. The elasticity of substitution indicates that for both factors of production the required changes are at the level of about 0.42%.

The significance of explanatory variables most often appeared in models accounting for the workload of nurses. And the best fit measured with the residual standard error was characterised by models accounting for the number of beds, the worst—the cost of outsourced services. Adjustments by explanatory variables in the discussed production function are shown in Table 2.

**Table 2:** Comparison of adjustment of Cobb-Douglas two-input functions according to explanatory variables.

	Total number of beds	Materials	Electricity	Doctors	Nurses	Outsourced services
median residual standard error	0.1490	0.3249	0.4092	0.4010	0.4010	0.4107
min. residual standard error	0.1423	0.1429	0.1490	0.1423	0.1520	0.1530
max. residual standard error	0.1530	0.3261	0.4227	0.4335	0.4107	0.4335
mean number of significant parameters (excluding intercept)	1	1	1.3333	1	1.4	1.3333
median number of significant parameters (excluding intercept	1	1	1	1	1	1

Calculated based on the data from Polish Association of Employers of Powiat Hospitals.

For three-factor estimations of the Cobb-Douglas function, we present the interpretation of the model with nurses, outsourced services, and electricity (M19) (Table B in S2 Appendix). As above, in the interpretation we use the principle of *ceteris paribus* and disregard the standards which may be applicable in a specific healthcare system. In the case of the three-factor Cobb-Douglas function given in the M19 model, the marginal productivities of factors amounted to (for averages): 0.0233 (nurses), 2.2275 (electricity) and 0.1082 (outsourced services). This means that the increase in outlays of these variables by an additional unit would increase the capacity to serve patients from 0.0233 patient-day in the case of employment of nurses, up to 2.2275 in the case of electricity costs. For medians, these values are 0.0985 (outsourced services), 0.2358 (electricity) and 0.0367 (nurses). In both cases, the largest changes in patient-days would result from a unit increase in electricity costs, and the smallest from an increase in the employment of nurses of 1 FTE. The same relationships occur in the production growth rates determined for individual factors. Patient service capacity in patient-days will increase by about 0.002% if the costs of outsourced services rises by a unit, and by 0.0351%-0.0478% if electricity costs rise by a unit and 0.0004%-0.0007% in the case of increase in the employment of nurses by 1 FTE.

A one-percent increase in the cost of outsourced services is related to an increase in patient-days by about 0.27%, in the case of a one-percent increase in electricity, the increase in patient-days is about 0.35%, and in case of the change in nurses’ FTEs by 0.14%. As in the case of the two-factor function discussed earlier, the benefit from economies of scale does not exceed 1, which means that increasing the outlay of all factors by 1% will result in an increase in the capacity to serve patients by less than 1%. The marginal substitution rate calculated for the nurses and electricity indicates that the reduction of workload of nurses by 1 FTE would require an increase in electricity outlay of 0.011–0.016 unit while maintaining the same level of patient-days. At first glance, this interpretation may seem irrational, because the reduction of FTE should result in a reduction in costs, but it should be remembered that despite this reduction, the number of patient-days should remain at the same level. This may mean, for example, the need for additional medical procedures for patients, which is connected with higher electricity costs. The same reduction in the FTEs would require, *ceteris paribus*, an increase in the costs of outsourced services by about 0.21–0.37 units, while reducing electricity costs by a unit would require, *ceteris paribus*, an increase in the costs of outsourced services of 20.58–23.94 units. A one-percent reduction in the workload of nurses would require, *ceteris paribus*, either a 0.399% increase in electricity costs or an 0.514% increase in the cost of outsourced services; and for the same percentage reduction in electricity costs, it would be necessary to increase the cost of outsourced services by nearly 1.3%.

The median residual standard error is the lowest for models in which total number of beds is used as an explanatory variable. Differences in median residual standard error for all explanatory variables except for the total number of beds are relatively small. Significant variables were most often found in models which included nurses, doctors, electricity costs and costs of outsourced services. Adjustments by explanatory variables for the discussed production function are shown in Table 3.

**Table 3:** Comparison of adjustment of three-factor Cobb-Douglas function by explanatory variables.

	Total number of beds	Materials	Electricity	Doctors	Nurses	Outsourced services
median residual standard error	0.1500	0.3063	0.3166	0.3115	0.3312	0.3063
min. residual standard error	0.1429	0.1429	0.1450	0.1429	0.1480	0.1474
max. residual standard error	0.1567	0.3367	0.4105	0.4105	0.3950	0.4105
mean number of significant parameters (excluding intercept)	1.4	1.3333	1.7778	1.7	1.9	1.6667
median number of significant parameters (excluding intercept	1	1	2	1.5	2	2

Calculated based on the data from Polish Association of Employers of Powiat Hospitals.

Despite the reservations raised above in relation to the results obtained for the two-input translog function, to make the considerations complete, its interpretation and analysis of the significance of variables and residual standard error are presented in this part due to the variables included in the model, similar to those of the Cobb-Douglas type function.

In the case of the two-factor translog function, we present the interpretation on the example of the M10 model, in which the explanatory variables are nurses’ workload and material costs. For this model, a relatively low residual standard error value was obtained, and also estimates of nearly all parameters remained significantly different from zero. The results presented in Table C in S2 Appendix indicate that marginal productivity of nurses’ FTEs, for averages and medians are 0.119 and 0.244 respectively. This means that increasing the employment of nurses by 1 FTE would enable the increase in patient service of 0.119–0.244 patient-day, with other factors unchanged. Increasing the employment of nurses by 1% would result in an increase in patient service by 0.48% (calculations for averages) and by 0.82% (for medians).

As indicated by the values of the marginal product of the cost of materials, increasing these costs by a unit would result, *ceteris paribus*, in increased patient service by approximately 0.26 and 0.08 (for averages and medians, respectively). On the other hand, an increase in the level of material costs by 1% would result in an increase in patient service by 0.24% as calculated for averages and by 0.08% for medians. The determined growth rates lead to the conclusion that the patient service capacity calculated in patient-days will increase by about 0.0013% and 0.0043% for averages and medians, respectively if the number of nurse FTEs rises by 1. The corresponding values for the cost of materials amount to 0.0028% and 0.0013%.

In the case of the discussed function, like in the Cobb-Douglas functions interpreted before, the benefits from the economies of scale do not exceed 1, which means that a 1% increase in the input of both factors will result in a relatively smaller increase in the number of patient-days. The marginal substitution rate indicates the need to increase material costs by 0.45–3.22 (for averages and medians respectively), in the event of the liquidation of 1 nurse FTE and the desire to maintain patient service at the current level. In the case of a 1% reduction in the workload of nurses, the change in the cost of materials required to maintain the current number of patient-days is, for averages, close to 2%, and for medians 10.196%.

Table 4 compares the significance of variables and residual standard error by factors of production. It can be noted that the significance of explanatory variables most often appeared in models accounting for the workload of nurses. On the other hand, the greatest fit measured with the residual standard error was characterised by models accounting for the number of beds, and the lowest adjustment was recorded for models accounting for the workload of doctors.

**Table 4:** Comparison of adjustment two-factor translog function by explanatory variables.

	Total number of beds	Materials	Electricity	Doctors	Nurses	Outsourced services
median residual standard error	0.1466	0.272	0.3102	0.3416	0.2907	0.2907
min. residual standard error	0.1412	0.1497	0.1466	0.1412	0.1461	0.1535
max. residual standard error	0.1535	0.3416	0.414	0.4176	0.3247	0.4176
mean number of significant parameters (excluding intercept)	1.2	1.6667	2.3333	0.8	3.2	1.3333
median number of significant parameters (excluding intercept	1	1	3	1	4	0

Calculated based on the data from Polish Association of Employers of Powiat Hospitals.

Below, there is an interpretation of the estimations of the three-factor translog function, in which the explanatory variables are: the workload of nurses and the costs of outsourced services and electricity (M19). On the basis of the marginal productivity of the factors (for averages) shown in Table D in S2 Appendix, it can be concluded that an increase in the workload of nurses by 1 FTE would entail an increase in the capacity of patient service by about 0.08 patient-day. If the cost of outsourced services or electricity were increased by a unit, it would be about 0.1 or 2.74, respectively. If the case of change of outlays of subsequent factors of 1%, *ceteris paribus*, the increases in the number of patient-days would amount to 0.3641 (nurses), 0.3198 (electricity) and 0.1783 (outsourced services). The values calculated for medians vary, from 0.0358 to 0.718 in the case of elasticity (percentage change in the number of patient-days caused by a one-percent change in the input of a given factor) to 0.014–1.58 in the case of marginal productivity (change in the number of patient-days caused by a unit change in the outlay of a given factor).

Production growth rates are highest for electricity costs, which means that an increase in these costs by a unit will enable the largest (in terms of percentages) increase in the number of patient-days.

Like in the case of the two-factor function, the benefits from the economies of scale do not exceed 1, and therefore an increase in the outlays of all factors by 1% will result in the increase in patient-days below 1 percent.

The marginal substitution rate calculated for the workload of nurse and costs of electricity indicates that a reduction of 1 FTE would entail an increase in the costs of electricity of 0.0299–0.1276 (for averages and medians, respectively), In the case of substitution of the workload of nurses by the cost of outsourced services, the obtained substitution rates indicate the need to increase the cost by 0.8 for averages or 14.56 units for medians which is a relatively large discrepancy compared to the results of calculations obtained with regard to the previous pair of production factors. And in the case of reducing the cost of electricity by a unit, increase in the costs of outsourced services should amount to from 28.65 unit for averages to 114.06 unit for medians.

Analogous relationships can be seen when considering changes of 1%. For example, calculating on the basis of averages, one percent reduction in FTEs would require, *ceteris paribus*, either an increase in electricity costs by 1.14%, or an increase in costs of outsourced services by 2.04%. In the case of the same percentage reduction in electricity costs, it would be necessary to increase costs of outsourced services by 1.8%. After determining these changes based on medians, we will get 3.27%, 20.07% and 6.14%, respectively.

Among the estimations of the three-factor translog models, the best fit was once again characterised by models accounting for the total number of beds. Significant variables were found most commonly in models which included nurses and electricity costs. Adjustments by explanatory variables for this function are included in Table 5.

**Table 5:** Comparison of adjustment of three-factor translog function by explanatory variables.

	Total number of beds	Materials	Electricity	Doctors	Nurses	Outsourced services
median residual standard error	0.1429	0.2512	0.2535	0.2749	0.2524	0.2512
min. residual standard error	0.1320	0.1385	0.1320	0.1320	0.1404	0.1341
max. residual standard error	0.1534	0.3221	0.3352	0.3352	0.2973	0.3352
mean number of significant parameters (excluding intercept)	1.2000	2.3333	3.4444	2.3000	3.3000	2.3333
median number of significant parameters (excluding intercept)	1	2	3	2.5	3	2

Calculated based on the data from Polish Association of Employers of Powiat Hospitals.

An interesting problem for the interpretation of results and the formulation of conclusions based on them is the question the impact on them of the assumptions necessary for the analytical tool, which is the production function. Thus, the obtained result shows the overlap of both the actual determinants in which the hospital operates as well as the analytical assumptions to be met to follow the requirements of production function.

The study presented in the paper has several limitations. First of all, as mentioned previously, the dataset we includes hospitals of different types (both public and non-public hospitals, with an without an Emergency Department or an Intensive Care Unit in their structure) which may lead to the OLS estimates being biased. Firstly, imposing other conditions on the dataset would result in the number of observations decreasing rapidly. When we include only relatively big (i.e. hospitals which have the number of beds between the first and the third quartile) public hospitals with an Emergency Department and an Intensive Care Unit in order to guarantee the greatest similarity of the units in the study, the number of hospitals is falls to 23. In our opinion this number of observations is too low to provide an important insight. Secondly, one of our goals has been to show the properties of production function which may prove useful in the analysis of hospitals and their functioning. Table 6 presents comparison of adjustment of two- and three-factor translog and Cobb-Douglas functions estimated by OLS on the subsample of 23 hospitals of similar characteristics by explanatory variables. Apart from some differences, especially in case of the number of significant variables, these results lead to similar conclusions to the ones discussed previously.

**Table 6:** Comparison of adjustment of two- and three-factor Cobb-Douglas and translog functions by explanatory variables (OLS, subsample).

	Total number of beds	Materials	Electricity	Doctors	Nurses	Outsourced services
Two-factor Cobb-Douglas function						
median residual standard error	0.1125	0.2527	0.2809	0.2774	0.2793	0.2774
min. residual standard error	0.1119	0.113	0.1131	0.1119	0.1125	0.1122
max. residual standard error	0.1131	0.2555	0.2847	0.2809	0.2847	0.2793
mean number of significant parameters (excluding intercept)	1	1	1	1	1	1
median number of significant parameters (excluding intercept)	1	1	1	1	1	1
Three-factor Cobb-Douglas function						
median residual standard error	0.1149	0.258	0.2587	0.2584	0.2597	0.2589
min. residual standard error	0.1124	0.1144	0.1147	0.1124	0.1139	0.1124
max. residual standard error	0.1159	0.2621	0.2878	0.2878	0.2878	0.2865
mean number of significant parameters (excluding intercept)	1	1	1	1	1	1
median number of significant parameters (excluding intercept)	1	1	1	1	1	1
Two-factor translog function						
median residual standard error	0.1181	0.2549	0.2246	0.2523	0.2268	0.2268
min. residual standard error	0.1154	0.1209	0.12	0.1163	0.1154	0.1181
max. residual standard error	0.1209	0.2638	0.2523	0.2638	0.2549	0.2527
mean number of significant parameters (excluding intercept)	0	0	1.6667	0.4	1	0.6667
median number of significant parameters (excluding intercept)	0	0	2	0	0	0
Three-factor translog function						
median residual standard error	0.1243	0.2279	0.2220	0.2263	0.2188	0.2155
min. residual standard error	0.1146	0.1146	0.1232	0.1183	0.1146	0.1220
max. residual standard error	0.1360	0.2700	0.2700	0.2700	0.2527	0.2374
mean number of significant parameters (excluding intercept)	0.0000	0.4444	0.5556	0.0000	0.5000	0.1111
median number of significant parameters (excluding intercept)	0	0	0	0	0	0

Calculated based on the data from Polish Association of Employers of Powiat Hospitals.

For all the models the total number of beds contributed to the best fit measured with residual standard error, which is in line with the results reported previously for the sample of 94 hospitals. For the two- and three-factor Cobb-Douglas functions the mean and median significance of explanatory variables was equal to 1 in all cases. In case of the translog models, significant variables were found most commonly in models which included nurses and electricity costs. This result is also similar to the one drawn for the whole sample of 94 hospitals.

Aiming for the consistency of this paper, we do not intend to analyse differences in estimates between various groups of hospitals which is an interesting and important sphere of future analysis. We believe, that introducing dynamics or implementing panel models in the future studies will provide much needed additional insight, especially in the light of the healthcare system reform which took place in Poland in 2017.

In the current study it was important for us to analyse, which inputs are mostly useful for the modelling. We compare however the consistency of our results with the results achieved implementing robust regression and regression on the smaller subset of hospitals with similar characteristics (relatively big public hospitals with an Emergency Department and an Intensive Care Unit within their structures), finding little differences.

## Supporting information

S1 Appendix Estimation results. (DOCX)

S2 Appendix Additional sources of knowledge. (DOCX)

## References

[1] OECD/European Union. *Health at a Glance*: *Europe 2020*: *State of Health in the EU Cycle*. Paris: OECD Publishing; 2020.

[2] Feldstein MS. *Economic analysis for health service efficiency*: *Econometric studies of the British National Health Service (Contributions to economic analysis)*. North-Holland Pub. Co; 1967.

[3] Stilwell JA. A Production Function Model of the Maternity Hospital. In: van Eimeren W, Engelbrecht R, Flagle CD, editors. *Third International Conference on System Science in Health Care*; 1984, pp 542–545; https://link.springer.com/content/pdf/10.1007/978-3-642-69939-9_123.pdf

[4] Lavers RJ, Whynes DK. A production function analysis of English maternity hospitals. Socio-Economics Planning Sciences 1978;12(2):85–93, https://www.sciencedirect.com/science/article/abs/pii/0038012178900058?via%3Dihub doi: 10.1016/0038-0121(78)90005-8 10308562

[5] Street A, Scheller-Kreinsen D, Geissler A, Busse R. (2010) *Determinants of hospital costs and performance variation*: *Methods*, *models and variables for the EuroDRG project*. Working Papers in Health Policy and Management, vol. 3. 2010 [October 22, 2021] https://www.researchgate.net/publication/44131706_Determinants_of_Hospital_Costs_and_Performance_Variation_Methods_Models_and_Variables_for_the_EuroDRG_Project/link/09e41509b72489e9e4000000/download

[6] Sanitas FR, Cadarso-Suarez C, Rodriguez_Alvarez MX. Estimating hospital production functions through flexible regression models. Mathematical and Computer Modelling 2011;54:1760–1764.

[7] Rosko MD, Broyles RW. *The Economics of Health Care*: *A Reference Handbook*. New York, Westport, CT: Greenwood Press, Inc.; 1988.

[8] McGuire A. The measurement of hospital efficiency. Social Science and Medicine 1987;24:719–724. doi: 10.1016/0277-9536(87)90108-0 3110969

[9] Grassetti L, Gori E, Minotti SC. Multilevel flexible specification of the production function in health economics. *IMA Journal of Management Mathematics* 2005;16:383–398.

[10] Li T, Rosenman R. Estimating hospital costs with a generalized Leontief function. *Health Econ*. 2001;10:523–538. doi: 10.1002/hec.605 11550293

[11] Liang YW, Chen WY, Lin YH. Estimating A Hospital Production Function To Evaluate The Effect Of Nurse Staffing On Patient Mortality In Taiwan: The Longitudinal Count Data Approach. Romanian Journal of Economic Forecasting–XVIII (4), 2015. Available from: http://www.ipe.ro/rjef/rjef4_15/rjef4_2015p154-169.pdf

[12] Reyes-Santías F, Cordova-Arevalo O, Rivo-Lopez E. Using flexible regression models for calculating hospital’s production functions. BMC Health Services Research 2020;20(1):641. doi: 10.1186/s12913-020-05465-2 32650764 PMC7350712

[13] Agarwal N, Ashlagi I, Azevedo E, Featherstone C, Karaduman Ö. *What Matters for the Productivity of Kidney Exchange*? AEA Papers and Proceedings MAY 2018, Vol. 108, PAPERS AND PROCEEDINGS OF THE One Hundred Thirtieth Annual Meeting OF THE AMERICAN ECONOMIC ASSOCIATION (MAY 2018):334–340.

[14] Karagiannis G, Tzouvelekas V. A Flexible Time-Varying Specification of the Technical Inefficiency Effects Model. Empirical Economics 2007;33(3):531–540.

[15] Battese GE, Coelli TJ. A Model for Technical Inefficiency Effects in a Stochastic Frontier Production Function for Panel Data. Empirical Economics 1995;20:325–332.

[16] Rosko MD, Mutter LD. Stochastic Frontier Analysis of Hospital Inefficiency A Review of Empirical Issues and an Assessment of Robustness. Medical Care Research and Review 2008;65(2):131–66. doi: 10.1177/1077558707307580 18045984

[17] Folland S, Hofler R. How reliable are hospital efficiency estimates? Exploiting the dual to homothetic production. *Health Economics* 2001 10(8):683–698. doi: 10.1002/hec.600 11747051

[18] Yildiz MS, Heboyan V, Khan MM. Estimating technical efficiency of Turkish hospitals: implications for hospital reform initiatives. BMC Health Service Research 2018;18:401 doi: 10.1186/s12913-018-3239-y 29866154 PMC5987422

[19] Frohloff A. (2007), *Cost and Technical Efficiency of German Hospitals—a Stochastic Frontier Analysis*. Ruhr Economic Papers no 2.

[20] Medin E, Anthun KS, Häkkinen U, Kittelsen SAC, Linna M, Magnussen J, et al. Cost efficiency of university hospitals in the Nordic countries: a cross-country analysis. The European Journal of Health Economics 2011;12(6):509–519. doi: 10.1007/s10198-010-0263-1 20668907

[21] Hamidi S. Measuring efficiency of governmental hospitals in Palestine using stochastic frontier analysis. *Cost* Eff Resour Alloc 2016;14:3. doi: 10.1186/s12962-016-0052-5 26848283 PMC4741008

[22] World Health Organization. The world health report: health systems financing: the path to universal coverage. World Health Organization; 2010. https://apps.who.int/iris/handle/10665/44371 10.2471/BLT.10.078741 PMC2878164 20539847

[23] Lave JR, Lave LB. Hospital cost functions. Ann. Rev. Public Health. 1984;5:193–213. doi: 10.1146/annurev.pu.05.050184.001205 6426487

[24] Hollingsworth B. Non-parametric and parametric applications measuring efficiency in health care. Health Care Management Science 2003;6:203–218. doi: 10.1023/a:1026255523228 14686627

[25] Hollingsworth B, Street A. The market for efficiency analysis of health care organizations. Health Economics 2006;15:1055–1059. doi: 10.1002/hec.1169 16991208

[26] Hollingsworth B. The measurement of efficiency and productivity of health care delivery. Health Economics 2008;17:1107–1128. doi: 10.1002/hec.1391 18702091

[27] Rosko MD, Mutter RL. What have we learned from the application of stochastic frontier analysis to U.S. hospitals. Medical Care Research and Review 2011;68(1 suppl):75S–100S. doi: 10.1177/1077558710370686 20519428

[28] Colombi R, Martini G, Vittandini G. Determinants of transient and persistent hospital efficiency: The case of Italy. Health Economics 2017;26(S2):5–22. doi: 10.1002/hec.3557 28940917

[29] Felt-Fisk S. New hospital information technology: Is it helping to improve quality? Issue Brief, 3 (May 2006) (www.mathematica-mpr.com/publications/pdfs/newhospinfo. Pdf).

[30] Raghupathi W, Tan J. Strategic uses of information technology in health care: A state-of-the-art survey. Topics in Health Information Management, 1999;20(1):1–15. 10539419

[31] Menon NM, Ulku Yaylacicegi U, Cezar A. Differential Effects of the Two Types of Information Systems: A Hospital-Based Study. Journal of Management Information Systems 2009;26(1):297–316.

[32] Lee J, McCullough JS, Town RJ. The impact of health information technology on hospital productivity. The RAND Journal of Economics 2013;44(3):545–568.

[33] Stadhouders N, Koolman X, van Dijk C, Jeurissen P, Adang E. The marginal benefits of healthcare spending in the Netherlands: Estimating cost-effectiveness thresholds using a translog production function. Health Econ. 2019;28(11):1331–1344. doi: 10.1002/hec.3946 31469510 PMC6851736

[34] Pourmohammadi K, Hatam N, Bastani P, Lotfi F. Estimating production function: a tool for Hospital Resource Management. Shiraz E-Med J. 2014;15(4):e23068. doi: 10.17795/semj23068

[35] Mehraban S, Raghfar H. Estimation of Production Function of Direct Health Care Services Delivered by Iranian Social Security Organization. International Journal of Hospital Research 2016;5(2):46–51.

[36] Mohammadi H, Meskarpour-Amiri M. Estimation Production Function of Inpatient Services and Input Productivity: A Cross-Sectional Study of Iran Selected Public Hospitals. Hospital Practices and Research 2016;1(3):91–93.

[37] Olsen KR, Gyrd-Hansen D, Sørensen TH, Kristensen T, Vedsted P, Street A. Organisational determinants of production and efficiency in general practice: a population-based study. The European Journal of Health Economics 2013;14(2):267–276. doi: 10.1007/s10198-011-0368-1 22143360

[38] Jensen GA, Morridey MA. The role of physicians in hospital production. The Review of Economics and Statistics 1986;68(3):432–442. doi: 10.1016/0167-6296(86)90017-2 10279034

[39] Grieco PE, McDevitt RC. Productivity and Quality in Health Care: Evidence from the Dialysis Industry. Review of Economic Studies 2017;84:1071–1105.

[40] Skinner J, Staiger D. Technology Diffusion and Productivity Growth in Health Care. The Review of Economics and Statistics 2015;97(5):951–964. doi: 10.1162/REST_a_00535 26989267 PMC4792131

[41] Morikawa M. *Economies of Scale and Hospital Productivity*: *An Empirical Analysis of Medical Area Level Panel Data*. RIETI Discussion Paper Series 10-E-050, October 2010, https://www.rieti.go.jp/jp/publications/dp/10e050.pdf

[42] Chen M-S, Lin C, Lee C-C. The production model of the administration organization system of the general hospital. Journal of Statistics and Management Systems 2003;6(3):463–477.

[43] Chiang AC. *Podstawy ekonomii matematycznej*. Warszawa: PWE; 2005.

[44] Simon CP, Blume L. *Mathematics for Economists*. NORTON;1994.

[45] Intriligator MD. *Mathematical Optimization and Economic Theory*. Prentice Hall; 1971.

[46] Henderson JM, Quandt RE. *A Microeconomic Theory A Mathematical Approach*. McGraw-Hill; 1980.

[47] R Core Team (2020). R: A language and environment for statistical computing. R Foundation for Statistical Computing, Vienna, Austria. URL https://www.R-project.org/

[48] Venables WN, Ripley BD. *Modern Applied Statistics with S*. Fourth Edition. New York: Springer; 2002.

