# Supplementary material for: Production function for modeling hospital activities. The case of Polish county hospitals
Source: PLoS One. 2022 May 12;17(5):e0268350. doi: 10.1371/journal.pone.0268350 (PMC9098024; doi:10.1371/journal.pone.0268350)
Supplement: S1 Appendix — (DOCX) [file pone.0268350.s001.docx]

S1 Appendix. Estimation results.

Table A. The results of the estimation of two-factor Cobb-Douglas function

|  | Estimate | Standard error | t | Pr(>\|t\|) | Residual standard error |
| --- | --- | --- | --- | --- | --- |
| M1 model | | | | | |
| Intercept | -1.8377 | 0.1989 | -9.2403 | 0.0000 | 0.1429 |
| ln(Total number of beds) | 1.0601 | 0.0555 | 19.1170 | 0.0000 |  |
| ln(Materials) | 0.0169 | 0.0352 | 0.4815 | 0.6313 |  |
| M2 model | | | | | |
| Intercept | -1.7890 | 0.1944 | -9.2014 | 0.0000 | 0.1490 |
| ln(Total number of beds) | 1.0527 | 0.0433 | 24.3231 | 0.0000 |  |
| ln(Electricity) | 0.0295 | 0.0321 | 0.9172 | 0.3615 |  |
| M3 model | | | | | |
| Intercept | -1.8925 | 0.1709 | -11.0719 | 0.0000 | 0.1423 |
| ln(Total number of beds) | 1.0815 | 0.0343 | 31.5470 | 0.0000 |  |
| ln(Doctors) | 0.0022 | 0.0160 | 0.1363 | 0.8919 |  |
| M4 model | | | | | |
| Intercept | -1.9225 | 0.1678 | -11.4553 | 0.0000 | 0.1520 |
| ln(Total number of beds) | 1.0463 | 0.0360 | 29.0315 | 0.0000 |  |
| ln(Nurses) | 0.0425 | 0.0215 | 1.9749 | 0.0513 |  |
| M5 model | | | | | |
| Intercept | -1.8525 | 0.1682 | -11.0120 | 0.0000 | 0.1530 |
| ln(Total number of beds) | 1.1256 | 0.0448 | 25.1246 | 0.0000 |  |
| ln(Outsourced services) | -0.0545 | 0.0391 | -1.3923 | 0.1672 |  |
| M6 model | | | | | |
| Intercept | 1.5903 | 0.2040 | 7.7938 | 0.0000 | 0.3249 |
| ln(Doctors) | 0.0348 | 0.0373 | 0.9337 | 0.3529 |  |
| ln(Materials) | 0.5371 | 0.0507 | 10.5991 | 0.0000 |  |
| M7 model | | | | | |
| Intercept | 2.5238 | 0.1732 | 14.5734 | 0.0000 | 0.4227 |
| ln(Doctors) | 0.0644 | 0.0422 | 1.5241 | 0.1309 |  |
| ln(Electricity) | 0.5533 | 0.0672 | 8.2298 | 0.0000 |  |
| M8 model | | | | | |
| Intercept | 1.1638 | 0.2950 | 3.9446 | 0.0002 | 0.4010 |
| ln(Doctors) | -0.0313 | 0.0618 | -0.5075 | 0.6130 |  |
| ln(Nurses) | 0.5516 | 0.0790 | 6.9864 | 0.0000 |  |
| M9 model | | | | | |
| Intercept | 0.6067 | 0.3976 | 1.5262 | 0.1304 | 0.4335 |
| ln(Doctors) | 0.0557 | 0.0477 | 1.1673 | 0.2461 |  |
| ln(Outsourced services) | 0.6295 | 0.0893 | 7.0495 | 0.0000 |  |
| M10 model | | | | | |
| Intercept | 1.3420 | 0.2461 | 5.4520 | 0.0000 | 0.3261 |
| ln(Nurses) | 0.1020 | 0.0517 | 1.9744 | 0.0514 |  |
| ln(Materials) | 0.4984 | 0.0549 | 9.0766 | 0.0000 |  |
| M11 model | | | | | |
| Intercept | 1.8618 | 0.2492 | 7.4721 | 0.0000 | 0.4092 |
| ln(Nurses) | 0.2019 | 0.0511 | 3.9494 | 0.0002 |  |
| ln(Electricity) | 0.4807 | 0.0636 | 7.5534 | 0.0000 |  |
| M12 model | | | | | |
| Intercept | 0.3486 | 0.4102 | 0.8499 | 0.3976 | 0.4107 |
| ln(Nurses) | 0.1857 | 0.0637 | 2.9168 | 0.0045 |  |
| ln(Outsourced services) | 0.5261 | 0.0932 | 5.6452 | 0.0000 |  |

Calculated based on the data from Polish Association of Employers of Powiat Hospitals.

Table B. The results of the estimation of three-factor Cobb-Douglas function

|  | Variable code | Estimate | Standard error | t | Pr(>\|t\|) | Residual standard error |
| --- | --- | --- | --- | --- | --- | --- |
| M1 model | | | | | | |
| Intercept |  | -1.7823 | 0.2049 | -8.6966 | 0.0000 | 0.1450 |
| ln(Total number of beds) | x1 | 1.0485 | 0.0562 | 18.6675 | 0.0000 |  |
| ln(Materials) | x2 | 0.0051 | 0.0382 | 0.1334 | 0.8941 |  |
| ln(Electricity) | x3 | 0.0271 | 0.0352 | 0.7698 | 0.4434 |  |
| M2 model | | | | | | |
| Intercept |  | -1.8380 | 0.2008 | -9.1523 | 0.0000 | 0.1429 |
| ln(Total number of beds) | x1 | 1.0601 | 0.0562 | 18.8802 | 0.0000 |  |
| ln(Materials) | x2 | 0.0168 | 0.0361 | 0.4672 | 0.6415 |  |
| ln(Doctors) | x3 | 0.0001 | 0.0165 | 0.0087 | 0.9931 |  |
| M3 model | | | | | | |
| Intercept |  | -1.9137 | 0.2001 | -9.5632 | 0.0000 | 0.1526 |
| ln(Total number of beds) | x1 | 1.0431 | 0.0551 | 18.9388 | 0.0000 |  |
| ln(Materials) | x2 | 0.0026 | 0.0358 | 0.0736 | 0.9415 |  |
| ln(Nurses) | x3 | 0.0421 | 0.0225 | 1.8743 | 0.0641 |  |
| M4 model | | | | | | |
| Intercept |  | -1.6381 | 0.2077 | -7.8882 | 0.0000 | 0.1474 |
| ln(Total number of beds) | x1 | 1.0724 | 0.0556 | 19.3040 | 0.0000 |  |
| ln(Materials) | x2 | 0.0596 | 0.0391 | 1.5237 | 0.1311 |  |
| ln(Outsourced services) | x3 | -0.0895 | 0.0446 | -2.0042 | 0.0481 |  |
| M5 model | | | | | | |
| Intercept |  | -1.7859 | 0.1969 | -9.0711 | 0.0000 | 0.1495 |
| ln(Total number of beds) | x1 | 1.0530 | 0.0441 | 23.8849 | 0.0000 |  |
| ln(Electricity) | x2 | 0.0304 | 0.0335 | 0.9062 | 0.3672 |  |
| ln(Doctors) | x3 | -0.0015 | 0.0166 | -0.0928 | 0.9262 |  |
| M6 model | | | | | | |
| Intercept |  | -1.8518 | 0.1955 | -9.4716 | 0.0000 | 0.1480 |
| ln(Total number of beds) | x1 | 1.0282 | 0.0455 | 22.6083 | 0.0000 |  |
| ln(Electricity) | x2 | 0.0207 | 0.0323 | 0.6393 | 0.5242 |  |
| ln(Nurses) | x3 | 0.0396 | 0.0220 | 1.7973 | 0.0756 |  |
| M7 model | | | | | | |
| Intercept |  | -1.6712 | 0.1989 | -8.4033 | 0.0000 | 0.1567 |
| ln(Total number of beds) | x1 | 1.0904 | 0.0502 | 21.7224 | 0.0000 |  |
| ln(Electricity) | x2 | 0.0482 | 0.0333 | 1.4487 | 0.1509 |  |
| ln(Outsourced services) | x3 | -0.0729 | 0.0412 | -1.7672 | 0.0806 |  |
| M8 model | | | | | | |
| Intercept |  | -1.9375 | 0.1721 | -11.2559 | 0.0000 | 0.1565 |
| ln(Total number of beds) | x1 | 1.0358 | 0.0366 | 28.2856 | 0.0000 |  |
| ln(Doctors) | x2 | -0.0453 | 0.0218 | -2.0820 | 0.0402 |  |
| ln(Nurses) | x3 | 0.0912 | 0.0299 | 3.0511 | 0.0030 |  |
| M9 model | | | | | | |
| Intercept |  | -1.8475 | 0.1690 | -10.9310 | 0.0000 | 0.1506 |
| ln(Total number of beds) | x1 | 1.1227 | 0.0452 | 24.8233 | 0.0000 |  |
| ln(Doctors) | x2 | 0.0092 | 0.0162 | 0.5654 | 0.5732 |  |
| ln(Outsourced services) | x3 | -0.0600 | 0.0405 | -1.4820 | 0.1418 |  |
| M10 model | | | | | | |
| Intercept |  | -1.8802 | 0.1677 | -11.2120 | 0.0000 | 0.1511 |
| ln(Total number of beds) | x1 | 1.0966 | 0.0459 | 23.8697 | 0.0000 |  |
| ln(Nurses) | x2 | 0.0536 | 0.0220 | 2.4302 | 0.0171 |  |
| ln(Outsourced services) | x3 | -0.0755 | 0.0401 | -1.8855 | 0.0626 |  |
| M11 model | | | | | | |
| Intercept |  | 1.7158 | 0.2120 | 8.0949 | 0.0000 | 0.3166 |
| ln(Doctors) | x1 | 0.0154 | 0.0382 | 0.4030 | 0.6879 |  |
| ln(Materials) | x2 | 0.4249 | 0.0689 | 6.1688 | 0.0000 |  |
| ln(Electricity) | x3 | 0.2003 | 0.0815 | 2.4565 | 0.0159 |  |
| M12 model | | | | | | |
| Intercept |  | 1.3134 | 0.2536 | 5.1788 | 0.0000 | 0.3349 |
| ln(Doctors) | x1 | -0.0270 | 0.0507 | -0.5330 | 0.5953 |  |
| ln(Materials) | x2 | 0.4987 | 0.0554 | 8.9992 | 0.0000 |  |
| ln(Nurses) | x3 | 0.1278 | 0.0705 | 1.8132 | 0.0731 |  |
| M13 model | | | | | | |
| Intercept |  | 1.4691 | 0.3347 | 4.3891 | 0.0000 | 0.3063 |
| ln(Doctors) | x1 | 0.0315 | 0.0390 | 0.8086 | 0.4209 |  |
| ln(Materials) | x2 | 0.5129 | 0.0766 | 6.6998 | 0.0000 |  |
| ln(Outsourced services) | x3 | 0.0474 | 0.1067 | 0.4445 | 0.6577 |  |
| M14 model | | | | | | |
| Intercept |  | 1.7820 | 0.2578 | 6.9114 | 0.0000 | 0.3709 |
| ln(Doctors) | x1 | -0.1098 | 0.0547 | -2.0072 | 0.0477 |  |
| ln(Electricity) | x2 | 0.5030 | 0.0654 | 7.6915 | 0.0000 |  |
| ln(Nurses) | x3 | 0.2927 | 0.0702 | 4.1680 | 0.0001 |  |
| M15 model | | | | | | |
| Intercept |  | 1.3458 | 0.3687 | 3.6503 | 0.0004 | 0.4105 |
| ln(Doctors) | x1 | 0.0106 | 0.0426 | 0.2487 | 0.8041 |  |
| ln(Electricity) | x2 | 0.3755 | 0.0789 | 4.7577 | 0.0000 |  |
| ln(Outsourced services) | x3 | 0.3604 | 0.0929 | 3.8782 | 0.0002 |  |
| M16 model | | | | | | |
| Intercept |  | 0.2840 | 0.4324 | 0.6569 | 0.5129 | 0.3919 |
| ln(Doctors) | x1 | -0.0490 | 0.0657 | -0.7460 | 0.4576 |  |
| ln(Outsourced services) | x2 | 0.5349 | 0.0964 | 5.5508 | 0.0000 |  |
| ln(Nurses) | x3 | 0.2273 | 0.0879 | 2.5855 | 0.0113 |  |
| M17 model | | | | | | |
| Intercept |  | 1.4604 | 0.2442 | 5.9804 | 0.0000 | 0.3367 |
| ln(Nurses) | x1 | 0.0929 | 0.0503 | 1.8469 | 0.0680 |  |
| ln(Materials) | x2 | 0.3870 | 0.0706 | 5.4814 | 0.0000 |  |
| ln(Electricity) | x3 | 0.1884 | 0.0772 | 2.4411 | 0.0166 |  |
| M18 model | | | | | | |
| Intercept |  | 1.2494 | 0.3481 | 3.5891 | 0.0005 | 0.3275 |
| ln(Nurses) | x1 | 0.0992 | 0.0529 | 1.8760 | 0.0639 |  |
| ln(Materials) | x2 | 0.4794 | 0.0777 | 6.1731 | 0.0000 |  |
| ln(Outsourced services) | x3 | 0.0375 | 0.1042 | 0.3602 | 0.7195 |  |
| M19 model | | | | | | |
| Intercept |  | 1.0981 | 0.3657 | 3.0025 | 0.0035 | 0.3950 |
| ln(Nurses) | x1 | 0.1411 | 0.0536 | 2.6322 | 0.0100 |  |
| ln(Electricity) | x2 | 0.3538 | 0.0741 | 4.7729 | 0.0000 |  |
| ln(Outsourced services) | x3 | 0.2746 | 0.0910 | 3.0176 | 0.0033 |  |

Calculated based on the data from Polish Association of Employers of Powiat Hospitals.

Table C. The results of the estimation of two-factor translog function

|  | Variable code | Estimate | Standard error | t | Pr(>\|t\|) | Residual standard error |
| --- | --- | --- | --- | --- | --- | --- |
| M1 model | | | | | | |
| (Intercept) |  | 2.3760 | 1.6808 | 1.4136 | 0.1610 | 0.1497 |
| ln(Total number of beds) | x1 | -0.9289 | 0.9279 | -1.0010 | 0.3196 |  |
| ln(Materials) | x2 | 0.5267 | 0.5426 | 0.9707 | 0.3343 |  |
| I(ln(Total number of beds)^2) |  | 0.2269 | 0.1427 | 1.5894 | 0.1156 |  |
| I(ln(Materials)^2) |  | 0.0044 | 0.0614 | 0.0711 | 0.9435 |  |
| I(ln(Total number of beds)*ln(Materials)) |  | -0.1029 | 0.1773 | -0.5802 | 0.5633 |  |
| M2 model | | | | | | |
| (Intercept) |  | 4.2253 | 1.5127 | 2.7932 | 0.0064 | 0.1466 |
| ln(Total number of beds) | x1 | -1.5855 | 0.6663 | -2.3794 | 0.0195 |  |
| ln(Electricity) | x2 | 1.0268 | 0.4406 | 2.3304 | 0.0221 |  |
| I(ln(Total number of beds)^2) |  | 0.2802 | 0.0754 | 3.7182 | 0.0004 |  |
| I(ln(Electricity)^2) |  | -0.0029 | 0.0488 | -0.0587 | 0.9534 |  |
| I(ln(Total number of beds)*ln(Electricity)) |  | -0.1802 | 0.1049 | -1.7183 | 0.0893 |  |
| M3 model | | | | | | |
| (Intercept) |  | 1.1855 | 1.2871 | 0.9211 | 0.3595 | 0.1412 |
| ln(Total number of beds) | x1 | -0.3446 | 0.5249 | -0.6566 | 0.5132 |  |
| ln(Doctors) | x2 | 0.3223 | 0.2049 | 1.5727 | 0.1194 |  |
| I(ln(Total number of beds)^2) |  | 0.1596 | 0.0571 | 2.7946 | 0.0064 |  |
| I(ln(Doctors)^2) |  | 0.0041 | 0.0058 | 0.7163 | 0.4757 |  |
| I(ln(Total number of beds)*ln(Doctors)) |  | -0.0654 | 0.0388 | -1.6837 | 0.0958 |  |
| M4 model | | | | | | |
| (Intercept) |  | 0.4965 | 1.3151 | 0.3775 | 0.7067 | 0.1461 |
| ln(Total number of beds) | x1 | -0.5566 | 0.5377 | -1.0351 | 0.3034 |  |
| ln(Nurses) | x2 | 0.7163 | 0.2489 | 2.8775 | 0.0050 |  |
| I(ln(Total number of beds)^2) |  | 0.1814 | 0.0689 | 2.6328 | 0.0100 |  |
| I(ln(Nurses)^2) |  | -0.0244 | 0.0253 | -0.9669 | 0.3362 |  |
| I(ln(Total number of beds)*ln(Nurses)) |  | -0.0683 | 0.0606 | -1.1265 | 0.2630 |  |
| M5 model | | | | | | |
| (Intercept) |  | 1.4965 | 1.3215 | 1.1324 | 0.2605 | 0.1535 |
| ln(Total number of beds) | x1 | -0.0837 | 0.7082 | -0.1182 | 0.9061 |  |
| ln(Outsourced services) | x2 | -0.1116 | 0.4693 | -0.2379 | 0.8125 |  |
| I(ln(Total number of beds)^2) |  | 0.1227 | 0.1032 | 1.1890 | 0.2376 |  |
| I(ln(Outsourced services)^2) |  | 0.0173 | 0.0590 | 0.2934 | 0.7699 |  |
| I(ln(Total number of beds)*ln(Outsourced services)) |  | -0.0213 | 0.1245 | -0.1714 | 0.8643 |  |
| M6 model | | | | | | |
| (Intercept) |  | 0.4320 | 0.9204 | 0.4693 | 0.6400 | 0.3416 |
| ln(Doctors) | x1 | 0.1275 | 0.2284 | 0.5582 | 0.5781 |  |
| ln(Materials) | x2 | 0.9977 | 0.3873 | 2.5759 | 0.0117 |  |
| I(ln(Doctors)^2) |  | 0.0039 | 0.0143 | 0.2747 | 0.7842 |  |
| I(ln(Materials)^2) |  | -0.0403 | 0.0566 | -0.7120 | 0.4784 |  |
| I(ln(Doctors)*ln(Materials)) |  | -0.0278 | 0.0569 | -0.4894 | 0.6257 |  |
| M7 model | | | | | | |
| (Intercept) |  | 2.7492 | 0.4170 | 6.5922 | 0.0000 | 0.4140 |
| ln(Doctors) | x1 | -0.0698 | 0.1428 | -0.4888 | 0.6262 |  |
| ln(Electricity) | x2 | 0.6049 | 0.3509 | 1.7239 | 0.0882 |  |
| I(ln(Doctors)^2) |  | 0.0219 | 0.0215 | 1.0194 | 0.3108 |  |
| I(ln(Electricity)^2) |  | 0.0177 | 0.1040 | 0.1707 | 0.8649 |  |
| I(ln(Doctors)*ln(Electricity)) |  | -0.0292 | 0.0796 | -0.3669 | 0.7145 |  |
| M8 model | | | | | | |
| (Intercept) |  | -8.9941 | 1.1122 | -8.0869 | 0.0000 | 0.3247 |
| ln(Doctors) | x1 | -0.4625 | 0.3424 | -1.3508 | 0.1802 |  |
| ln(Nurses) | x2 | 4.4980 | 0.5146 | 8.7406 | 0.0000 |  |
| I(ln(Doctors)^2) |  | 0.0249 | 0.0299 | 0.8335 | 0.4068 |  |
| I(ln(Nurses)^2) |  | -0.3765 | 0.0694 | -5.4212 | 0.0000 |  |
| I(ln(Doctors)*ln(Nurses)) |  | 0.0600 | 0.0859 | 0.6988 | 0.4865 |  |
| M9 model | | | | | | |
| (Intercept) |  | 3.0593 | 2.1839 | 1.4008 | 0.1648 | 0.4176 |
| ln(Doctors) | x1 | 0.2538 | 0.6086 | 0.4171 | 0.6776 |  |
| ln(Outsourced services) | x2 | -0.5796 | 1.0242 | -0.5659 | 0.5729 |  |
| I(ln(Doctors)^2) |  | -0.0049 | 0.0180 | -0.2751 | 0.7839 |  |
| I(ln(Outsourced services)^2) |  | 0.1408 | 0.1382 | 1.0189 | 0.3110 |  |
| I(ln(Doctors)*ln(Outsourced services)) |  | -0.0334 | 0.1211 | -0.2760 | 0.7832 |  |
| M10 model | | | | | | |
| (Intercept) |  | -4.2290 | 1.1119 | -3.8034 | 0.0003 | 0.2720 |
| ln(Nurses) | x1 | 2.8412 | 0.4632 | 6.1340 | 0.0000 |  |
| ln(Materials) | x2 | -0.7377 | 0.3934 | -1.8754 | 0.0641 |  |
| I(ln(Nurses)^2) |  | -0.3357 | 0.0530 | -6.3333 | 0.0000 |  |
| I(ln(Materials)^2) |  | -0.1351 | 0.0493 | -2.7418 | 0.0074 |  |
| I(ln(Nurses)*ln(Materials)) |  | 0.3669 | 0.0787 | 4.6607 | 0.0000 |  |
| M11 model | | | | | | |
| (Intercept) |  | -5.8385 | 1.0535 | -5.5419 | 0.0000 | 0.3102 |
| ln(Nurses) | x1 | 3.3615 | 0.3970 | 8.4665 | 0.0000 |  |
| ln(Electricity) | x2 | -1.0552 | 0.3363 | -3.1377 | 0.0023 |  |
| I(ln(Nurses)^2) |  | -0.3117 | 0.0395 | -7.8983 | 0.0000 |  |
| I(ln(Electricity)^2) |  | -0.1031 | 0.0786 | -1.3112 | 0.1932 |  |
| I(ln(Nurses)*ln(Electricity)) |  | 0.3168 | 0.0816 | 3.8831 | 0.0002 |  |
| M12 model | | | | | | |
| (Intercept) |  | -0.6222 | 1.5506 | -0.4013 | 0.6892 | 0.2907 |
| ln(Nurses) | x1 | 2.1258 | 0.4183 | 5.0821 | 0.0000 |  |
| ln(Outsourced services) | x2 | -1.3753 | 0.5978 | -2.3006 | 0.0238 |  |
| I(ln(Nurses)^2) |  | -0.3558 | 0.0425 | -8.3804 | 0.0000 |  |
| I(ln(Outsourced services)^2) |  | -0.1117 | 0.0940 | -1.1877 | 0.2382 |  |
| I(ln(Nurses)*ln(Outsourced services)) |  | 0.4936 | 0.1088 | 4.5357 | 0.0000 |  |

Calculated based on the data from Polish Association of Employers of Powiat Hospitals.

Table D. The results of the estimation of three-factor translog function

|  | Variable code | Estimate | Standard error | t | Pr(>\|t\|) | Residual standard error |  |
| --- | --- | --- | --- | --- | --- | --- | --- |
| M1 model | | | | | | | |
| (Intercept) |  | 4.2172 | 1.7124 | 2.4627 | 0.0158 | 0.1385 |  |
| ln(Total number of beds) | x1 | -1.4787 | 0.9503 | -1.5560 | 0.1235 |  |  |
| ln(Materials) | x2 | -0.1397 | 0.5819 | -0.2401 | 0.8108 |  |  |
| ln(Electricity) | x3 | 1.0382 | 0.4953 | 2.0962 | 0.0391 |  |  |
| I(ln(Total number of beds)^2) |  | 0.2480 | 0.1468 | 1.6892 | 0.0949 |  |  |
| I(ln(Materials)^2) |  | -0.0054 | 0.0656 | -0.0820 | 0.9349 |  |  |
| I(ln(Electricity)^2) |  | 0.0292 | 0.0738 | 0.3954 | 0.6936 |  |  |
| I(ln(Total number of beds)*ln(Materials)) |  | 0.0512 | 0.1778 | 0.2878 | 0.7742 |  |  |
| I(ln(Total number of beds)*ln(Electricity)) |  | -0.1640 | 0.1276 | -1.2850 | 0.2023 |  |  |
| I(ln(Electricity)*ln(Materials)) |  | -0.0523 | 0.1114 | -0.4692 | 0.6401 |  |  |
| M2 model | | | | | | | |
| (Intercept) |  | 2.4706 | 1.6509 | 1.4966 | 0.1383 | 0.1437 |  |
| ln(Total number of beds) | x1 | -1.0316 | 0.9082 | -1.1359 | 0.2592 |  |  |
| ln(Materials) | x2 | 0.5061 | 0.5463 | 0.9264 | 0.3569 |  |  |
| ln(Doctors) | x3 | 0.0793 | 0.2442 | 0.3250 | 0.7460 |  |  |
| I(ln(Total number of beds)^2) |  | 0.2197 | 0.1428 | 1.5391 | 0.1275 |  |  |
| I(ln(Materials)^2) |  | 0.0195 | 0.0598 | 0.3257 | 0.7454 |  |  |
| I(ln(Doctors)^2) |  | 0.0081 | 0.0062 | 1.3019 | 0.1965 |  |  |
| I(ln(Total number of beds)*ln(Materials)) |  | -0.0756 | 0.1771 | -0.4271 | 0.6704 |  |  |
| I(ln(Total number of beds)*ln(Doctors)) |  | 0.0188 | 0.0600 | 0.3136 | 0.7546 |  |  |
| I(ln(Doctors)*ln(Materials)) |  | -0.0591 | 0.0359 | -1.6464 | 0.1034 |  |  |
| M3 model | | | | | | | |
| (Intercept) |  | 0.7918 | 1.7582 | 0.4504 | 0.6536 | 0.1524 |  |
| ln(Total number of beds) | x1 | -1.0784 | 0.8996 | -1.1987 | 0.2340 |  |  |
| ln(Materials) | x2 | 0.2081 | 0.5716 | 0.3641 | 0.7167 |  |  |
| ln(Nurses) | x3 | 0.9468 | 0.4070 | 2.3263 | 0.0224 |  |  |
| I(ln(Total number of beds)^2) |  | 0.2702 | 0.1418 | 1.9054 | 0.0601 |  |  |
| I(ln(Materials)^2) |  | -0.0065 | 0.0612 | -0.1069 | 0.9151 |  |  |
| I(ln(Nurses)^2) |  | -0.0543 | 0.0355 | -1.5313 | 0.1295 |  |  |
| I(ln(Total number of beds)*ln(Materials)) |  | -0.0905 | 0.1760 | -0.5143 | 0.6084 |  |  |
| I(ln(Total number of beds)*ln(Nurses)) |  | -0.0816 | 0.0962 | -0.8486 | 0.3985 |  |  |
| I(ln(Nurses)*ln(Materials)) |  | 0.0554 | 0.0689 | 0.8039 | 0.4237 |  |  |
| M4 model | | | | | | | |
| (Intercept) |  | 3.8513 | 1.6873 | 2.2826 | 0.0250 | 0.1484 |  |
| ln(Total number of beds) | x1 | -0.9312 | 0.9447 | -0.9857 | 0.3271 |  |  |
| ln(Materials) | x2 | 1.4947 | 0.6544 | 2.2839 | 0.0249 |  |  |
| ln(Outsourced services) | x3 | -1.3724 | 0.6381 | -2.1507 | 0.0344 |  |  |
| I(ln(Total number of beds)^2) |  | 0.1011 | 0.1658 | 0.6096 | 0.5438 |  |  |
| I(ln(Materials)^2) |  | 0.1205 | 0.0802 | 1.5019 | 0.1369 |  |  |
| I(ln(Outsourced services)^2) |  | 0.0472 | 0.0638 | 0.7397 | 0.4615 |  |  |
| I(ln(Total number of beds)*ln(Materials)) |  | -0.2018 | 0.1726 | -1.1696 | 0.2455 |  |  |
| I(ln(Total number of beds)*ln(Outsourced services)) |  | 0.3620 | 0.1990 | 1.8188 | 0.0725 |  |  |
| I(ln(Outsourced services) *ln(Materials)) |  | -0.2741 | 0.1533 | -1.7872 | 0.0775 |  |  |
| M5 model | | | | | | | |
| (Intercept) |  | 3.7867 | 1.5233 | 2.4859 | 0.0149 | 0.1320 |  |
| ln(Total number of beds) | x1 | -1.3751 | 0.6904 | -1.9918 | 0.0496 |  |  |
| ln(Electricity) | x2 | 0.7899 | 0.4598 | 1.7179 | 0.0895 |  |  |
| ln(Doctors) | x3 | 0.0483 | 0.2298 | 0.2104 | 0.8339 |  |  |
| I(ln(Total number of beds)^2) |  | 0.2534 | 0.0833 | 3.0426 | 0.0031 |  |  |
| I(ln(Electricity)^2) |  | -0.0057 | 0.0582 | -0.0981 | 0.9221 |  |  |
| I(ln(Doctors)^2) |  | 0.0137 | 0.0085 | 1.6103 | 0.1111 |  |  |
| I(ln(Total number of beds)*ln(Electricity)) |  | -0.1041 | 0.1145 | -0.9088 | 0.3661 |  |  |
| I(ln(Total number of beds)*ln(Doctors)) |  | -0.0172 | 0.0437 | -0.3943 | 0.6944 |  |  |
| I(ln(Electricity)*ln(Doctors)) |  | -0.0378 | 0.0316 | -1.1946 | 0.2356 |  |  |
| M6 model | | | | | | | |
| (Intercept) |  | 2.6150 | 1.6026 | 1.6317 | 0.1065 | 0.1534 |  |
| ln(Total number of beds) | x1 | -1.6237 | 0.6985 | -2.3246 | 0.0225 |  |  |
| ln(Electricity) | x2 | 0.7457 | 0.4539 | 1.6427 | 0.1042 |  |  |
| ln(Nurses) | x3 | 0.7249 | 0.3292 | 2.2015 | 0.0304 |  |  |
| I(ln(Total number of beds)^2) |  | 0.3118 | 0.0934 | 3.3386 | 0.0013 |  |  |
| I(ln(Electricity)^2) |  | -0.0060 | 0.0532 | -0.1121 | 0.9110 |  |  |
| I(ln(Nurses)^2) |  | -0.0300 | 0.0285 | -1.0531 | 0.2953 |  |  |
| I(ln(Total number of beds)*ln(Electricity)) |  | -0.1670 | 0.1103 | -1.5138 | 0.1338 |  |  |
| I(ln(Total number of beds)*ln(Nurses)) |  | -0.0723 | 0.0681 | -1.0622 | 0.2912 |  |  |
| I(ln(Electricity)*ln(Nurses)) |  | 0.0382 | 0.0506 | 0.7547 | 0.4525 |  |  |
| M7 model | | | | | | | |
| (Intercept) |  | 4.0405 | 1.5199 | 2.6584 | 0.0094 | 0.1341 |  |
| ln(Total number of beds) | x1 | -0.7540 | 0.7976 | -0.9453 | 0.3472 |  |  |
| ln(Electricity) | x2 | 1.2638 | 0.4591 | 2.7527 | 0.0072 |  |  |
| ln(Outsourced services) | x3 | -0.9100 | 0.5250 | -1.7334 | 0.0867 |  |  |
| I(ln(Total number of beds)^2) |  | 0.0992 | 0.1247 | 0.7956 | 0.4285 |  |  |
| I(ln(Electricity)^2) |  | 0.0476 | 0.0580 | 0.8205 | 0.4142 |  |  |
| I(ln(Outsourced services)^2) |  | 0.0237 | 0.0596 | 0.3970 | 0.6924 |  |  |
| I(ln(Total number of beds)*ln(Electricity)) |  | -0.0782 | 0.1182 | -0.6614 | 0.5102 |  |  |
| I(ln(Total number of beds)*ln(Outsourced services)) |  | 0.1887 | 0.1406 | 1.3419 | 0.1832 |  |  |
| I(ln(Electricity)*ln(Outsourced services)) |  | -0.1948 | 0.0993 | -1.9626 | 0.0530 |  |  |
| M8 model | | | | | | | |
| (Intercept) |  | 0.8107 | 1.3302 | 0.6095 | 0.5439 | 0.1404 |  |
| ln(Total number of beds) | x1 | -0.4772 | 0.5389 | -0.8855 | 0.3784 |  |  |
| ln(Nurses) | x2 | 0.4922 | 0.4919 | 1.0006 | 0.3199 |  |  |
| ln(Doctors) | x3 | 0.0217 | 0.3959 | 0.0548 | 0.9564 |  |  |
| I(ln(Total number of beds)^2) |  | 0.1554 | 0.0701 | 2.2173 | 0.0293 |  |  |
| I(ln(Nurses)^2) |  | 0.0194 | 0.0388 | 0.4996 | 0.6187 |  |  |
| I(ln(Doctors)^2) |  | 0.0085 | 0.0136 | 0.6276 | 0.5320 |  |  |
| I(ln(Total number of beds)*ln(Nurses)) |  | -0.0605 | 0.0914 | -0.6614 | 0.5101 |  |  |
| I(ln(Total number of beds)*ln(Doctors)) |  | 0.0372 | 0.0644 | 0.5767 | 0.5657 |  |  |
| I(ln(Doctors)*ln(Nurses)) |  | -0.0603 | 0.0409 | -1.4760 | 0.1437 |  |  |
| M9 model | | | | | | | |
| (Intercept) |  | 1.4318 | 1.3695 | 1.0455 | 0.2988 | 0.1421 |  |
| ln(Total number of beds) | x1 | -0.0855 | 0.7297 | -0.1172 | 0.9070 |  |  |
| ln(Doctors) | x2 | 0.2972 | 0.2404 | 1.2363 | 0.2198 |  |  |
| ln(Outsourced services) | x3 | -0.3471 | 0.5296 | -0.6555 | 0.5139 |  |  |
| I(ln(Total number of beds)^2) |  | 0.1447 | 0.1073 | 1.3486 | 0.1811 |  |  |
| I(ln(Doctors)^2) |  | 0.0052 | 0.0062 | 0.8337 | 0.4068 |  |  |
| I(ln(Outsourced services)^2) |  | 0.0137 | 0.0708 | 0.1929 | 0.8475 |  |  |
| I(ln(Total number of beds)*ln(Doctors)) |  | -0.0908 | 0.0873 | -1.0401 | 0.3013 |  |  |
| I(ln(Total number of beds)*ln(Outsourced services)) |  | 0.0072 | 0.1311 | 0.0547 | 0.9565 |  |  |
| I(ln(Outsourced services)*ln(Doctors)) |  | 0.0308 | 0.0821 | 0.3752 | 0.7084 |  |  |
| M10 model | | | | | | | |
| (Intercept) |  | 0.6772 | 1.4250 | 0.4752 | 0.6359 | 0.1439 |  |
| ln(Total number of beds) | x1 | -0.3065 | 0.7371 | -0.4159 | 0.6786 |  |  |
| ln(Nurses) | x2 | 0.7653 | 0.2824 | 2.7097 | 0.0082 |  |  |
| ln(Outsourced services) | x3 | -0.3903 | 0.5103 | -0.7649 | 0.4465 |  |  |
| I(ln(Total number of beds)^2) |  | 0.1126 | 0.1527 | 0.7373 | 0.4630 |  |  |
| I(ln(Nurses)^2) |  | -0.0291 | 0.0347 | -0.8375 | 0.4047 |  |  |
| I(ln(Outsourced services)^2) |  | 0.0361 | 0.0632 | 0.5713 | 0.5693 |  |  |
| I(ln(Total number of beds)*ln(Nurses)) |  | -0.0123 | 0.1459 | -0.0843 | 0.9330 |  |  |
| I(ln(Total number of beds)*ln(Outsourced services)) |  | 0.0461 | 0.1753 | 0.2627 | 0.7934 |  |  |
| I(ln(Outsourced services)*ln(Nurses)) |  | -0.0550 | 0.1756 | -0.3133 | 0.7548 |  |  |
| M11 model | | | | | | | |
| (Intercept) |  | 0.3160 | 0.9776 | 0.3232 | 0.7473 | 0.3221 |  |
| ln(Doctors) | x1 | 0.3105 | 0.2890 | 1.0744 | 0.2857 |  |  |
| ln(Materials) | x2 | 0.3561 | 0.4729 | 0.7530 | 0.4536 |  |  |
| ln(Electricity) | x3 | 1.0409 | 0.4722 | 2.2043 | 0.0302 |  |  |
| I(ln(Doctors)^2) |  | -0.0023 | 0.0195 | -0.1169 | 0.9072 |  |  |
| I(ln(Materials)^2) |  | 0.1959 | 0.1008 | 1.9424 | 0.0554 |  |  |
| I(ln(Electricity)^2) |  | 0.4130 | 0.1702 | 2.4269 | 0.0174 |  |  |
| I(ln(Doctors)*ln(Materials)) |  | -0.0793 | 0.0794 | -0.9980 | 0.3211 |  |  |
| I(ln(Doctors)*ln(Electricity)) |  | 0.0398 | 0.0934 | 0.4261 | 0.6712 |  |  |
| I(ln(Electricity)*ln(Materials)) |  | -0.6290 | 0.2187 | -2.8755 | 0.0051 |  |  |
| M12 model | | | | | | | |
| (Intercept) |  | -4.2944 | 1.1977 | -3.5856 | 0.0006 | 0.2642 |  |
| ln(Doctors) | x1 | 0.0951 | 0.4125 | 0.2306 | 0.8182 |  |  |
| ln(Materials) | x2 | -0.8319 | 0.4004 | -2.0780 | 0.0408 |  |  |
| ln(Nurses) | x3 | 2.8510 | 0.6501 | 4.3857 | 0.0000 |  |  |
| I(ln(Doctors)^2) |  | 0.0316 | 0.0255 | 1.2381 | 0.2191 |  |  |
| I(ln(Materials)^2) |  | -0.1404 | 0.0497 | -2.8248 | 0.0059 |  |  |
| I(ln(Nurses)^2) |  | -0.3208 | 0.0745 | -4.3040 | 0.0000 |  |  |
| I(ln(Doctors)*ln(Materials)) |  | 0.0005 | 0.0716 | 0.0066 | 0.9947 |  |  |
| I(ln(Doctors)*ln(Nurses)) |  | -0.0618 | 0.0740 | -0.8356 | 0.4057 |  |  |
| I(ln(Nurses)*ln(Materials)) |  | 0.3887 | 0.1017 | 3.8208 | 0.0003 |  |  |
| M13 model | | | | | | | |
| (Intercept) |  | 0.0167 | 2.0393 | 0.0082 | 0.9935 | 0.3102 |  |
| ln(Doctors) | x1 | 0.3155 | 0.5509 | 0.5726 | 0.5684 |  |  |
| ln(Materials) | x2 | 1.3210 | 0.6293 | 2.0992 | 0.0388 |  |  |
| ln(Outsourced services) | x3 | -0.3151 | 1.0297 | -0.3060 | 0.7603 |  |  |
| I(ln(Doctors)^2) |  | 0.0018 | 0.0157 | 0.1124 | 0.9108 |  |  |
| I(ln(Materials)^2) |  | 0.0762 | 0.1177 | 0.6471 | 0.5194 |  |  |
| I(ln(Outsourced services)^2) |  | 0.1913 | 0.1698 | 1.1264 | 0.2632 |  |  |
| I(ln(Doctors)*ln(Materials)) |  | 0.0105 | 0.0903 | 0.1168 | 0.9073 |  |  |
| I(ln(Doctors)*ln(Outsourced services)) |  | -0.0647 | 0.1444 | -0.4479 | 0.6554 |  |  |
| I(ln(Outsourced services)*ln(Materials)) |  | -0.2999 | 0.2429 | -1.2351 | 0.2202 |  |  |
| M14 model | | | | | | | |
| (Intercept) |  | -5.9847 | 1.1109 | -5.3871 | 0.0000 | 0.2973 |  |
| ln(Doctors) | x1 | -0.0851 | 0.3327 | -0.2559 | 0.7987 |  |  |
| ln(Electricity) | x2 | -1.0393 | 0.3747 | -2.7737 | 0.0068 |  |  |
| ln(Nurses) | x3 | 3.4714 | 0.5217 | 6.6534 | 0.0000 |  |  |
| I(ln(Doctors)^2) |  | 0.0395 | 0.0372 | 1.0611 | 0.2917 |  |  |
| I(ln(Electricity)^2) |  | -0.1205 | 0.0818 | -1.4729 | 0.1445 |  |  |
| I(ln(Nurses)^2) |  | -0.3186 | 0.0689 | -4.6260 | 0.0000 |  |  |
| I(ln(Doctors)*ln(Electricity)) |  | -0.0325 | 0.0885 | -0.3676 | 0.7141 |  |  |
| I(ln(Doctors)*ln(Nurses)) |  | -0.0304 | 0.0816 | -0.3724 | 0.7105 |  |  |
| I(ln(Electricity)*ln(Nurses)) |  | 0.3542 | 0.1204 | 2.9410 | 0.0042 |  |  |
| M15 model | | | | | | | |
| (Intercept) |  | 1.1298 | 1.9430 | 0.5815 | 0.5625 | 0.3352 |  |
| ln(Doctors) | x1 | 0.1398 | 0.5082 | 0.2750 | 0.7840 |  |  |
| ln(Electricity) | x2 | 2.7030 | 0.6502 | 4.1570 | 0.0001 |  |  |
| ln(Outsourced services) | x3 | -0.6575 | 0.9156 | -0.7181 | 0.4747 |  |  |
| I(ln(Doctors)^2) |  | -0.0092 | 0.0202 | -0.4543 | 0.6508 |  |  |
| I(ln(Electricity)^2) |  | 0.3416 | 0.1368 | 2.4971 | 0.0145 |  |  |
| I(ln(Outsourced services)^2) |  | 0.2556 | 0.1256 | 2.0343 | 0.0451 |  |  |
| I(ln(Doctors)*ln(Electricity)) |  | 0.0088 | 0.0752 | 0.1167 | 0.9074 |  |  |
| I(ln(Doctors)*ln(Outsourced services)) |  | -0.0059 | 0.1040 | -0.0571 | 0.9546 |  |  |
| I(ln(Electricity)*ln(Outsourced services)) |  | -0.7482 | 0.1928 | -3.8816 | 0.0002 |  |  |
| M16 model | | | | | | | |
| (Intercept) |  | -0.7384 | 1.8897 | -0.3907 | 0.6970 | 0.2857 |  |
| ln(Doctors) | x1 | -0.2717 | 0.7008 | -0.3877 | 0.6992 |  |  |
| ln(Nurses) | x2 | 2.3098 | 0.7817 | 2.9549 | 0.0041 |  |  |
| ln(Outsourced services) | x3 | -1.2957 | 0.7037 | -1.8413 | 0.0691 |  |  |
| I(ln(Doctors)^2) |  | 0.0293 | 0.0310 | 0.9432 | 0.3483 |  |  |
| I(ln(Nurses)^2) |  | -0.3456 | 0.0682 | -5.0706 | 0.0000 |  |  |
| I(ln(Outsourced services)^2) |  | -0.1393 | 0.1063 | -1.3111 | 0.1934 |  |  |
| I(ln(Doctors)*ln(Nurses)) |  | -0.0504 | 0.0834 | -0.6042 | 0.5474 |  |  |
| I(ln(Doctors)*ln(Outsourced services)) |  | 0.0596 | 0.1275 | 0.4674 | 0.6415 |  |  |
| I(ln(Outsourced services)*ln(Nurses)) |  | 0.4796 | 0.1511 | 3.1737 | 0.0021 |  |  |
| M17 model | | | | | | | |
| (Intercept) |  | -3.3122 | 1.2785 | -2.5906 | 0.0113 | 0.2613 |  |
| ln(Nurses) | x1 | 2.4403 | 0.5012 | 4.8687 | 0.0000 |  |  |
| ln(Materials) | x2 | -1.1027 | 0.4995 | -2.2074 | 0.0300 |  |  |
| ln(Electricity) | x3 | 0.9874 | 0.5297 | 1.8641 | 0.0658 |  |  |
| I(ln(Nurses)^2) |  | -0.2912 | 0.0539 | -5.3968 | 0.0000 |  |  |
| I(ln(Materials)^2) |  | -0.0174 | 0.0917 | -0.1897 | 0.8500 |  |  |
| I(ln(Electricity)^2) |  | 0.3181 | 0.1308 | 2.4330 | 0.0171 |  |  |
| I(ln(Nurses)*ln(Materials)) |  | 0.3796 | 0.1194 | 3.1803 | 0.0021 |  |  |
| I(ln(Nurses)*ln(Electricity)) |  | -0.1013 | 0.1283 | -0.7899 | 0.4318 |  |  |
| I(ln(Electricity)*ln(Materials)) |  | -0.3734 | 0.1786 | -2.0911 | 0.0395 |  |  |
| M18 model | | | | | | | |
| (Intercept) |  | -1.8033 | 1.7479 | -1.0317 | 0.3052 | 0.2512 |  |
| ln(Nurses) | x1 | 2.3394 | 0.6619 | 3.5343 | 0.0007 |  |  |
| ln(Materials) | x2 | 0.5402 | 0.7496 | 0.7207 | 0.4731 |  |  |
| ln(Outsourced services) | x3 | -1.5925 | 0.8073 | -1.9725 | 0.0518 |  |  |
| I(ln(Nurses)^2) |  | -0.3616 | 0.0554 | -6.5336 | 0.0000 |  |  |
| I(ln(Materials)^2) |  | 0.0009 | 0.1068 | 0.0081 | 0.9935 |  |  |
| I(ln(Outsourced services)^2) |  | 0.1719 | 0.1151 | 1.4931 | 0.1392 |  |  |
| I(ln(Nurses)*ln(Materials)) |  | 0.2353 | 0.1253 | 1.8771 | 0.0640 |  |  |
| I(ln(Nurses)*ln(Outsourced services)) |  | 0.2727 | 0.2126 | 1.2827 | 0.2031 |  |  |
| I(ln(Outsourced services)*ln(Materials)) |  | -0.3548 | 0.2044 | -1.7354 | 0.0863 |  |  |
| M19 model | | | | | | | |
| (Intercept) |  | -1.0402 | 1.6865 | -0.6168 | 0.5391 | 0.2535 |  |
| ln(Nurses) | x1 | 1.7305 | 0.4995 | 3.4644 | 0.0008 |  |  |
| ln(Electricity) | x2 | 1.7245 | 0.5664 | 3.0449 | 0.0031 |  |  |
| ln(Outsourced services) | x3 | -1.3969 | 0.6344 | -2.2021 | 0.0304 |  |  |
| I(ln(Nurses)^2) |  | -0.3190 | 0.0408 | -7.8229 | 0.0000 |  |  |
| I(ln(Electricity)^2) |  | 0.2080 | 0.0948 | 2.1946 | 0.0309 |  |  |
| I(ln(Outsourced services)^2) |  | 0.0271 | 0.0946 | 0.2870 | 0.7748 |  |  |
| I(ln(Nurses)*ln(Electricity)) |  | 0.0870 | 0.0851 | 1.0214 | 0.3100 |  |  |
| I(ln(Nurses)*ln(Outsourced services)) |  | 0.4387 | 0.1208 | 3.6316 | 0.0005 |  |  |
| I(ln(Electricity)*ln(Outsourced services)) |  | -0.5674 | 0.1300 | -4.3655 | 0.0000 |  |  |

Calculated based on the data from Polish Association of Employers of Powiat Hospitals.
