# Supplementary material for: Production function for modeling hospital activities. The case of Polish county hospitals
Source: PLoS One. 2022 May 12;17(5):e0268350. doi: 10.1371/journal.pone.0268350 (PMC9098024; doi:10.1371/journal.pone.0268350)
Supplement: S2 Appendix — (DOCX) [file pone.0268350.s002.docx]

S2 Appendix. Additional sources of knowledge.

Table A. Additional sources of knowledge resulting from the two-factor Cobb-Douglas function

| Model | Variable | Marginal productivity (for means) | Marginal productivity (for medians) | Production growth rate (for means) | Production growth rate (for medians) | Marginal rate of technical substitution (for means) | Marginal rate of technical substitution (for medians) | Elasticity of substitution (for means) | Elasticity of substitution (for medians) |
| --- | --- | --- | --- | --- | --- | --- | --- | --- | --- |
| M1 | x1=Total number of beds | 0.2535 | 0.2505 | 0.0043 | 0.0047 | 21.8227 | 16.6324 | 62.6220 | 62.6220 |
|  | x2=Materials | 0.0116 | 0.0151 | 0.0002 | 0.0003 |  |  |  |  |
| M2 | x1=Total number of beds | 0.2519 | 0.2483 | 0.0042 | 0.0047 | 1.4512 | 1.1709 | 35.7042 | 35.7042 |
|  | x2=Electricity | 0.1736 | 0.2121 | 0.0029 | 0.0040 |  |  |  |  |
| M3 | x1=Total number of beds | 0.2581 | 0.2557 | 0.0044 | 0.0048 | 243.5633 | 123.1980 | 496.0919 | 496.0919 |
|  | x2=Doctors | 0.0011 | 0.0021 | 0.0000 | 0.0000 |  |  |  |  |
| M4 | x1=Total number of beds | 0.2544 | 0.2458 | 0.0042 | 0.0046 | 38.1285 | 20.7018 | 24.6384 | 24.6384 |
|  | x2=Nurses | 0.0067 | 0.0119 | 0.0001 | 0.0002 |  |  |  |  |
| M5 | x1=Total number of beds | 0.2675 | 0.2667 | 0.0045 | 0.0050 | -13.4148 | -12.5895 | -20.6618 | -20.6618 |
|  | x2=Outsourced services | -0.0199 | -0.0212 | -0.0003 | -0.0004 |  |  |  |  |
| M6 | x1=Doctors | 0.0182 | 0.0316 | 0.0003 | 0.0006 | 0.0460 | 0.0694 | 0.0649 | 0.0649 |
|  | x2=Materials | 0.3953 | 0.4559 | 0.0062 | 0.0090 |  |  |  |  |
| M7 | x1=Doctors | 0.0323 | 0.0562 | 0.0005 | 0.0011 | 0.0096 | 0.0154 | 0.1164 | 0.1164 |
|  | x2=Electricity | 3.3505 | 3.6595 | 0.0549 | 0.0748 |  |  |  |  |
| M8 | x1=Doctors | -0.0189 | -0.0285 | -0.0003 | -0.0006 | -0.1791 | -0.1922 | -0.0568 | -0.0568 |
|  | x2=Nurses | 0.1055 | 0.1483 | 0.0014 | 0.0029 |  |  |  |  |
| M9 | x1=Doctors | 0.0269 | 0.0506 | 0.0005 | 0.0010 | 0.1170 | 0.2171 | 0.0885 | 0.0885 |
|  | x2=Outsourced services | 0.2297 | 0.2333 | 0.0039 | 0.0046 |  |  |  |  |
| M10 | x1=Nurses | 0.0172 | 0.0271 | 0.0003 | 0.0005 | 0.0461 | 0.0647 | 0.2047 | 0.2047 |
|  | x2=Materials | 0.3738 | 0.4180 | 0.0058 | 0.0083 |  |  |  |  |
| M11 | x1=Nurses | 0.0342 | 0.0517 | 0.0005 | 0.0011 | 0.0110 | 0.0164 | 0.4200 | 0.4200 |
|  | x2=Electricity | 3.0993 | 3.1562 | 0.0477 | 0.0650 |  |  |  |  |
| M12 | x1=Nurses | 0.0300 | 0.0490 | 0.0005 | 0.0010 | 0.1481 | 0.2560 | 0.3530 | 0.3530 |
|  | x2=Outsourced services | 0.2026 | 0.1915 | 0.0033 | 0.0038 |  |  |  |  |

Calculated based on the data from Polish Association of Employers of Powiat Hospitals.

Table B. Additional sources of knowledge resulting from the three-factor Cobb-Douglas function

| Model | Variable | Marginal productivity (for means) | Marginal productivity (for medians) | Production growth rate (for means) | Production growth rate (for medians) | Pair of variables | Marginal rate of technical substitution (for means) | Marginal rate of technical substitution (for medians) | Elasticity of substitution (for means) | Elasticity of substitution (for medians) |
| --- | --- | --- | --- | --- | --- | --- | --- | --- | --- | --- |
| M1 | x1=Total number of beds | 0.251 | 0.2473 | 0.0042 | 0.0046 | (x1,x2) | 71.7516 | 54.6864 | 205.8969 | 205.8969 |
|  | x2=Materials | 0.0035 | 0.0045 | 0.0001 | 0.0001 | (x1,x3) | 1.5746 | 1.2704 | 38.7388 | 38.7388 |
|  | x3=Electricity | 0.1594 | 0.1947 | 0.0027 | 0.0037 | (x2,x3) | 0.0219 | 0.0232 | 0.1881 | 0.1881 |
| M2 | x1=Total number of beds | 0.2535 | 0.2505 | 0.0043 | 0.0047 | (x1,x2) | 21.9359 | 16.7187 | 62.9468 | 62.9468 |
|  | x2=Materials | 0.0116 | 0.015 | 0.0002 | 0.0003 | (x1,x3) | 3621.6134 | 1831.867 | 7376.534 | 7376.534 |
|  | x3=Doctors | 0.0001 | 0.0001 | 0 | 0 | (x2,x3) | 165.0997 | 109.5697 | 117.1867 | 117.1867 |
| M3 | x1=Total number of beds | 0.2536 | 0.245 | 0.0042 | 0.0046 | (x1,x2) | 138.0393 | 105.2084 | 396.1145 | 396.1145 |
|  | x2=Materials | 0.0018 | 0.0023 | 0 | 0 | (x1,x3) | 38.3449 | 20.8193 | 24.7783 | 24.7783 |
|  | x3=Nurses | 0.0066 | 0.0118 | 0.0001 | 0.0002 | (x2,x3) | 0.2778 | 0.1979 | 0.0626 | 0.0626 |
| M4 | x1=Total number of beds | 0.2573 | 0.2536 | 0.0043 | 0.0048 | (x1,x2) | 6.2697 | 4.7785 | 17.9913 | 17.9913 |
|  | x2=Materials | 0.041 | 0.0531 | 0.0007 | 0.001 | (x1,x3) | -7.7823 | -7.3036 | -11.9866 | -11.9866 |
|  | x3=Outsourced services | -0.0331 | -0.0347 | -0.0006 | -0.0007 | (x2,x3) | -1.2413 | -1.5284 | -0.6662 | -0.6662 |
| M5 | x1=Total number of beds | 0.2517 | 0.2484 | 0.0042 | 0.0047 | (x1,x2) | 1.4096 | 1.1373 | 34.6805 | 34.6805 |
|  | x2=Electricity | 0.1786 | 0.2184 | 0.003 | 0.0041 | (x1,x3) | -334.9874 | -169.442 | -682.305 | -682.305 |
|  | x3=Doctors | -0.0008 | -0.0015 | 0 | 0 | (x2,x3) | -237.6464 | -148.985 | -19.674 | -19.674 |
| M6 | x1=Total number of beds | 0.2503 | 0.2412 | 0.0041 | 0.0046 | (x1,x2) | 2.0234 | 1.6326 | 49.7827 | 49.7827 |
|  | x2=Electricity | 0.1237 | 0.1477 | 0.002 | 0.0028 | (x1,x3) | 40.2266 | 21.8409 | 25.9942 | 25.9942 |
|  | x3=Nurses | 0.0062 | 0.011 | 0.0001 | 0.0002 | (x2,x3) | 19.8803 | 13.3783 | 0.5222 | 0.5222 |
| M7 | x1=Total number of beds | 0.2605 | 0.2574 | 0.0044 | 0.0048 | (x1,x2) | 0.9187 | 0.7413 | 22.6038 | 22.6038 |
|  | x2=Electricity | 0.2835 | 0.3472 | 0.0048 | 0.0065 | (x1,x3) | -9.714 | -9.1164 | -14.9618 | -14.9618 |
|  | x3=Outsourced services | -0.0268 | -0.0282 | -0.0005 | -0.0005 | (x2,x3) | -10.5732 | -12.2985 | -0.6619 | -0.6619 |
| M8 | x1=Total number of beds | 0.2516 | 0.2435 | 0.0042 | 0.0046 | (x1,x2) | -11.2228 | -5.6767 | -22.8587 | -22.8587 |
|  | x2=Doctors | -0.0224 | -0.0429 | -0.0004 | -0.0008 | (x1,x3) | 17.5769 | 9.5433 | 11.3581 | 11.3581 |
|  | x3=Nurses | 0.0143 | 0.0255 | 0.0002 | 0.0005 | (x2,x3) | -1.5662 | -1.6812 | -0.4969 | -0.4969 |
| M9 | x1=Total number of beds | 0.2681 | 0.2657 | 0.0045 | 0.005 | (x1,x2) | 60.1415 | 30.4205 | 122.4967 | 122.4967 |
|  | x2=Doctors | 0.0045 | 0.0087 | 0.0001 | 0.0002 | (x1,x3) | -12.154 | -11.4063 | -18.72 | -18.72 |
|  | x3=Outsourced services | -0.0221 | -0.0233 | -0.0004 | -0.0004 | (x2,x3) | -0.2021 | -0.375 | -0.1528 | -0.1528 |
| M10 | x1=Total number of beds | 0.267 | 0.2579 | 0.0044 | 0.0049 | (x1,x2) | 31.6846 | 17.2031 | 20.4744 | 20.4744 |
|  | x2=Nurses | 0.0084 | 0.015 | 0.0001 | 0.0003 | (x1,x3) | -9.4249 | -8.8451 | -14.5165 | -14.5165 |
|  | x3=Outsourced services | -0.0283 | -0.0292 | -0.0005 | -0.0005 | (x2,x3) | -0.2975 | -0.5142 | -0.709 | -0.709 |
| M11 | x1=Doctors | 0.008 | 0.0138 | 0.0001 | 0.0003 | (x1,x2) | 0.0257 | 0.0388 | 0.0362 | 0.0362 |
|  | x2=Materials | 0.3112 | 0.3568 | 0.0049 | 0.0071 | (x1,x3) | 0.0064 | 0.0102 | 0.0769 | 0.0769 |
|  | x3=Electricity | 1.2577 | 1.3621 | 0.0199 | 0.0271 | (x2,x3) | 0.2474 | 0.2619 | 2.1215 | 2.1215 |
| M12 | x1=Doctors | -0.0143 | -0.0242 | -0.0002 | -0.0005 | (x1,x2) | -0.0385 | -0.058 | -0.0542 | -0.0542 |
|  | x2=Materials | 0.3727 | 0.4179 | 0.0058 | 0.0083 | (x1,x3) | -0.6668 | -0.7157 | -0.2115 | -0.2115 |
|  | x3=Nurses | 0.0215 | 0.0338 | 0.0003 | 0.0007 | (x2,x3) | 17.3362 | 12.3499 | 3.9039 | 3.9039 |
| M13 | x1=Doctors | 0.0164 | 0.0286 | 0.0003 | 0.0006 | (x1,x2) | 0.0436 | 0.0657 | 0.0614 | 0.0614 |
|  | x2=Materials | 0.376 | 0.4353 | 0.0059 | 0.0086 | (x1,x3) | 0.8785 | 1.63 | 0.6644 | 0.6644 |
|  | x3=Outsourced services | 0.0187 | 0.0175 | 0.0003 | 0.0003 | (x2,x3) | 20.1493 | 24.8106 | 10.815 | 10.815 |
| M14 | x1=Doctors | -0.0577 | -0.0951 | -0.0009 | -0.002 | (x1,x2) | -0.0181 | -0.0288 | -0.2184 | -0.2184 |
|  | x2=Electricity | 3.1932 | 3.2987 | 0.0499 | 0.068 | (x1,x3) | -1.1826 | -1.2694 | -0.3752 | -0.3752 |
|  | x3=Nurses | 0.0488 | 0.0749 | 0.0008 | 0.0015 | (x2,x3) | 65.4201 | 44.0239 | 1.7183 | 1.7183 |
| M15 | x1=Doctors | 0.0052 | 0.0095 | 0.0001 | 0.0002 | (x1,x2) | 0.0023 | 0.0037 | 0.0282 | 0.0282 |
|  | x2=Electricity | 2.2383 | 2.5438 | 0.0373 | 0.0508 | (x1,x3) | 0.0389 | 0.0722 | 0.0294 | 0.0294 |
|  | x3=Outsourced services | 0.1345 | 0.1314 | 0.0022 | 0.0026 | (x2,x3) | 16.6449 | 19.361 | 1.042 | 1.042 |
| M16 | x1=Doctors | -0.0248 | -0.0438 | -0.0004 | -0.0009 | (x1,x2) | -0.1211 | -0.2247 | -0.0916 | -0.0916 |
|  | x2=Outsourced services | 0.2044 | 0.1947 | 0.0033 | 0.0039 | (x1,x3) | -0.6795 | -0.7293 | -0.2156 | -0.2156 |
|  | x3=Nurses | 0.0364 | 0.06 | 0.0006 | 0.0012 | (x2,x3) | 5.6097 | 3.2454 | 2.3535 | 2.3535 |
| M17 | x1=Nurses | 0.0157 | 0.0244 | 0.0002 | 0.0005 | (x1,x2) | 0.054 | 0.0759 | 0.24 | 0.24 |
|  | x2=Materials | 0.2909 | 0.3217 | 0.0045 | 0.0065 | (x1,x3) | 0.0129 | 0.0192 | 0.4929 | 0.4929 |
|  | x3=Electricity | 1.2145 | 1.2688 | 0.0187 | 0.0255 | (x2,x3) | 0.2395 | 0.2536 | 2.0537 | 2.0537 |
| M18 | x1=Nurses | 0.0167 | 0.0263 | 0.0003 | 0.0005 | (x1,x2) | 0.0466 | 0.0654 | 0.207 | 0.207 |
|  | x2=Materials | 0.3585 | 0.402 | 0.0055 | 0.008 | (x1,x3) | 1.109 | 1.9169 | 2.6433 | 2.6433 |
|  | x3=Outsourced services | 0.0151 | 0.0137 | 0.0002 | 0.0003 | (x2,x3) | 23.7868 | 29.2896 | 12.7674 | 12.7674 |
| M19 | x1=Nurses | 0.0233 | 0.0367 | 0.0004 | 0.0007 | (x1,x2) | 0.0105 | 0.0156 | 0.3988 | 0.3988 |
|  | x2=Electricity | 2.2275 | 2.358 | 0.0351 | 0.0478 | (x1,x3) | 0.2156 | 0.3726 | 0.5138 | 0.5138 |
|  | x3=Outsourced services | 0.1082 | 0.0985 | 0.0017 | 0.002 | (x2,x3) | 20.5792 | 23.9372 | 1.2883 | 1.2883 |

Calculated based on the data from Polish Association of Employers of Powiat Hospitals.

Table C. Additional sources of knowledge resulting from the two-factor translog function

| Model | Variable | Marginal productivity (for means) | Marginal productivity (for medians) | Production growth rate (for means) | Production growth rate (for medians) | Elasticity (for means) | Elasticity (for medians) | Pair of variables | Marginal rate of technical substitution (for means) | Marginal rate of technical substitution (for medians) | Elasticity of substitution (for means) | Elasticity of substitution (for medians) |
| --- | --- | --- | --- | --- | --- | --- | --- | --- | --- | --- | --- | --- |
| M1 | x1=Total number of beds | 0.2599 | 0.2558 | 0.0045 | 0.0049 | 1.1141 | 1.1088 | (x1,x2) | -287.089 | 56.9702 | -823.823 | 214.4956 |
|  | x2=Materials | -0.0009 | 0.0045 | 0 | 0.0001 | -0.001 | 0.0052 |  |  |  |  |  |
| M2 | x1=Total number of beds | 0.2549 | 0.2511 | 0.0044 | 0.0048 | 1.0877 | 1.0904 | (x1,x2) | 2.2156 | 0.9213 | 54.5111 | 28.0925 |
|  | x2=Electricity | 0.1151 | 0.2726 | 0.002 | 0.0052 | 0.02 | 0.0388 |  |  |  |  |  |
| M3 | x1=Total number of beds | 0.2543 | 0.2561 | 0.0044 | 0.005 | 1.1011 | 1.1217 | (x1,x2) | 482.0065 | 293.2914 | 981.755 | 1181.022 |
|  | x2=Doctors | 0.0005 | 0.0009 | 0 | 0 | 0.0011 | 0.0009 |  |  |  |  |  |
| M4 | x1=Total number of beds | 0.2522 | 0.2423 | 0.0042 | 0.0047 | 1.0372 | 1.051 | (x1,x2) | 32.4799 | 9.7781 | 20.9884 | 11.6375 |
|  | x2=Nurses | 0.0078 | 0.0248 | 0.0001 | 0.0005 | 0.0494 | 0.0903 |  |  |  |  |  |
| M5 | x1=Total number of beds | 0.2681 | 0.262 | 0.0047 | 0.0051 | 1.161 | 1.1411 | (x1,x2) | -14.1627 | -12.2673 | -21.8139 | -20.133 |
|  | x2=Outsourced services | -0.0189 | -0.0214 | -0.0003 | -0.0004 | -0.053 | -0.0567 |  |  |  |  |  |
| M6 | x1=Doctors | 0.0222 | 0.0422 | 0.0003 | 0.0008 | 0.041 | 0.0451 | (x1,x2) | 0.0576 | 0.0868 | 0.0812 | 0.0812 |
|  | x2=Materials | 0.3844 | 0.4862 | 0.0058 | 0.0093 | 0.5048 | 0.5559 |  |  |  |  |  |
| M7 | x1=Doctors | 0.0357 | 0.0411 | 0.0006 | 0.0009 | 0.0731 | 0.0481 | (x1,x2) | 0.0111 | 0.0114 | 0.1337 | 0.0861 |
|  | x2=Electricity | 3.2301 | 3.6154 | 0.0542 | 0.0755 | 0.5465 | 0.5582 |  |  |  |  |  |
| M8 | x1=Doctors | 0.0995 | 0.0538 | 0.0011 | 0.0009 | 0.1336 | 0.0526 | (x1,x2) | 1.3762 | 0.2252 | 0.4366 | 0.0666 |
|  | x2=Nurses | 0.0723 | 0.2391 | 0.0008 | 0.0042 | 0.306 | 0.7906 |  |  |  |  |  |
| M9 | x1=Doctors | 0.0173 | 0.044 | 0.0003 | 0.0009 | 0.037 | 0.0499 | (x1,x2) | 0.0709 | 0.1822 | 0.0536 | 0.0743 |
|  | x2=Outsourced services | 0.244 | 0.2414 | 0.0043 | 0.0049 | 0.6909 | 0.6722 |  |  |  |  |  |
| M10 | x1=Nurses | 0.119 | 0.2443 | 0.0013 | 0.0043 | 0.4825 | 0.8218 | (x1,x2) | 0.4519 | 3.223 | 2.0068 | 10.196 |
|  | x2=Materials | 0.2634 | 0.0758 | 0.0028 | 0.0013 | 0.2405 | 0.0806 |  |  |  |  |  |
| M11 | x1=Nurses | 0.0896 | 0.213 | 0.001 | 0.0038 | 0.3843 | 0.7261 | (x1,x2) | 0.0286 | 0.1464 | 1.0878 | 3.7504 |
|  | x2=Electricity | 3.1374 | 1.455 | 0.0351 | 0.0262 | 0.3533 | 0.1936 |  |  |  |  |  |
| M12 | x1=Nurses | 0.0921 | 0.239 | 0.001 | 0.0043 | 0.4 | 0.8239 | (x1,x2) | 0.3937 | 5.2655 | 0.9383 | 7.2609 |
|  | x2=Outsourced services | 0.2339 | 0.0454 | 0.0026 | 0.0008 | 0.4263 | 0.1135 |  |  |  |  |  |

Calculated based on the data from Polish Association of Employers of Powiat Hospitals.

Table D. Additional sources of knowledge resulting from the three-factor translog function

| Model | Variable | Marginal productivity (for means) | Marginal productivity (for medians) | Production growth rate (for means) | Production growth rate (for medians) | Elasticity (for means) | Elasticity (for medians) | Pair of variables | Marginal rate of technical substitution (for means) | Marginal rate of technical substitution (for medians) | Elasticity of substitution (for means) | Elasticity of substitution (for medians) |
| --- | --- | --- | --- | --- | --- | --- | --- | --- | --- | --- | --- | --- |
| M1 | x1=Total number of beds | 0.258 | 0.2504 | 0.0045 | 0.0048 | 1.1052 | 1.0902 | (x1,x2) | -14.6483 | -26.2455 | -42.0343 | -98.8154 |
|  | x2=Materials | -0.0176 | -0.0095 | -0.0003 | -0.0002 | -0.026 | -0.011 | (x1,x3) | 1.2525 | 0.6811 | 30.8156 | 20.7694 |
|  | x3=Electricity | 0.206 | 0.3677 | 0.0036 | 0.0071 | 0.0359 | 0.0525 | (x2,x3) | -0.0855 | -0.026 | -0.7331 | -0.2102 |
| M2 | x1=Total number of beds | 0.2643 | 0.2545 | 0.0046 | 0.0049 | 1.1444 | 1.1158 | (x1,x2) | -19.0939 | 16.5559 | -54.7913 | 62.3339 |
|  | x2=Materials | -0.0138 | 0.0154 | -0.0002 | 0.0003 | -0.021 | 0.0179 | (x1,x3) | -216.685 | 59.2889 | -441.346 | 238.7437 |
|  | x3=Doctors | -0.0012 | 0.0043 | 0 | 0.0001 | -0.003 | 0.0047 | (x2,x3) | 11.3484 | 3.5811 | 8.055 | 3.8301 |
| M3 | x1=Total number of beds | 0.2546 | 0.2438 | 0.0041 | 0.0047 | 1.012 | 1.0515 | (x1,x2) | -17.6161 | -6.1112 | -50.5508 | -23.0088 |
|  | x2=Materials | -0.0145 | -0.0399 | -0.0002 | -0.0008 | -0.02 | -0.0457 | (x1,x3) | 16.1555 | 5.4855 | 10.4396 | 6.5286 |
|  | x3=Nurses | 0.0158 | 0.0444 | 0.0003 | 0.0009 | 0.0969 | 0.1611 | (x2,x3) | -0.9171 | -0.8976 | -0.2065 | -0.2837 |
| M4 | x1=Total number of beds | 0.2632 | 0.2586 | 0.0045 | 0.005 | 1.1226 | 1.1201 | (x1,x2) | 6.1256 | 7.8149 | 17.578 | 29.4233 |
|  | x2=Materials | 0.043 | 0.0331 | 0.0007 | 0.0006 | 0.0639 | 0.0381 | (x1,x3) | -6.0875 | -9.958 | -9.3762 | -16.343 |
|  | x3=Outsourced services | -0.0432 | -0.026 | -0.0007 | -0.0005 | -0.12 | -0.0685 | (x2,x3) | -0.9938 | -1.2742 | -0.5334 | -0.5554 |
| M5 | x1=Total number of beds | 0.2535 | 0.2496 | 0.0044 | 0.0048 | 1.0956 | 1.0932 | (x1,x2) | 5.3654 | 0.7026 | 132.0053 | 21.4245 |
|  | x2=Electricity | 0.0472 | 0.3552 | 0.0008 | 0.0069 | 0.0083 | 0.051 | (x1,x3) | -238.961 | -26.5602 | -486.717 | -106.952 |
|  | x3=Doctors | -0.0011 | -0.0094 | 0 | -0.0002 | -0.002 | -0.0102 | (x2,x3) | -44.5372 | -37.8033 | -3.6871 | -4.9921 |
| M6 | x1=Total number of beds | 0.2457 | 0.2409 | 0.004 | 0.0046 | 0.9986 | 1.0422 | (x1,x2) | 1.6465 | 1.9862 | 40.5091 | 60.5651 |
|  | x2=Electricity | 0.1492 | 0.1213 | 0.0024 | 0.0023 | 0.0247 | 0.0172 | (x1,x3) | 26.6357 | 9.1873 | 17.2119 | 10.9344 |
|  | x3=Nurses | 0.0092 | 0.0262 | 0.0002 | 0.0005 | 0.058 | 0.0953 | (x2,x3) | 16.1771 | 4.6256 | 0.4249 | 0.1805 |
| M7 | x1=Total number of beds | 0.2622 | 0.2511 | 0.0045 | 0.0048 | 1.118 | 1.0935 | (x1,x2) | 0.7237 | 0.501 | 17.8051 | 15.2762 |
|  | x2=Electricity | 0.3623 | 0.5013 | 0.0062 | 0.0097 | 0.0628 | 0.0716 | (x1,x3) | -9.148 | -14.9935 | -14.09 | -24.6072 |
|  | x3=Outsourced services | -0.0287 | -0.0167 | -0.0005 | -0.0003 | -0.079 | -0.0444 | (x2,x3) | -12.6406 | -29.9293 | -0.7913 | -1.6108 |
| M8 | x1=Total number of beds | 0.2553 | 0.2382 | 0.0043 | 0.0046 | 1.0548 | 1.0392 | (x1,x2) | 16.3904 | 6.9954 | 10.5914 | 8.3257 |
|  | x2=Nurses | 0.0156 | 0.0341 | 0.0003 | 0.0007 | 0.0996 | 0.1248 | (x1,x3) | -10.2363 | -10.4141 | -20.8493 | -41.9353 |
|  | x3=Doctors | -0.0249 | -0.0229 | -0.0004 | -0.0004 | -0.051 | -0.0248 | (x2,x3) | -0.6245 | -1.4887 | -1.9685 | -5.0369 |
| M9 | x1=Total number of beds | 0.2552 | 0.2629 | 0.0045 | 0.0051 | 1.1107 | 1.1526 | (x1,x2) | 213.4534 | -165.329 | 434.7637 | -665.746 |
|  | x2=Doctors | 0.0012 | -0.0016 | 0 | 0 | 0.0026 | -0.0017 | (x1,x3) | -34.3423 | -14.0754 | -52.895 | -23.1005 |
|  | x3=Outsourced services | -0.0074 | -0.0187 | -0.0001 | -0.0004 | -0.021 | -0.0499 | (x2,x3) | -0.1609 | 0.0851 | -0.1217 | 0.0347 |
| M10 | x1=Total number of beds | 0.2685 | 0.2474 | 0.0044 | 0.0048 | 1.0959 | 1.0759 | (x1,x2) | 23.4798 | 7.3443 | 15.1725 | 8.7409 |
|  | x2=Nurses | 0.0114 | 0.0337 | 0.0002 | 0.0006 | 0.0722 | 0.1231 | (x1,x3) | -7.3344 | -8.862 | -11.2967 | -14.5442 |
|  | x3=Outsourced services | -0.0366 | -0.0279 | -0.0006 | -0.0005 | -0.097 | -0.074 | (x2,x3) | -0.3124 | -1.2066 | -0.7446 | -1.6639 |
| M11 | x1=Doctors | 0.0135 | 0.0403 | 0.0002 | 0.0008 | 0.027 | 0.0473 | (x1,x2) | 0.0712 | 0.1325 | 0.1004 | 0.1239 |
|  | x2=Materials | 0.1896 | 0.3043 | 0.0031 | 0.0064 | 0.2689 | 0.3815 | (x1,x3) | 0.0067 | 0.0223 | 0.0804 | 0.169 |
|  | x3=Electricity | 2.0301 | 1.807 | 0.0333 | 0.0378 | 0.3358 | 0.2797 | (x2,x3) | 0.0934 | 0.1684 | 0.8007 | 1.3639 |
| M12 | x1=Doctors | 0.0257 | 0.0271 | 0.0003 | 0.0005 | 0.033 | 0.0273 | (x1,x2) | 0.1012 | 0.4908 | 0.1425 | 0.4589 |
|  | x2=Materials | 0.2537 | 0.0552 | 0.0027 | 0.001 | 0.2313 | 0.0595 | (x1,x3) | 0.2214 | 0.1117 | 0.0702 | 0.033 |
|  | x3=Nurses | 0.1159 | 0.2426 | 0.0012 | 0.0044 | 0.4693 | 0.8276 | (x2,x3) | 2.1883 | 0.2276 | 0.4928 | 0.0719 |
| M13 | x1=Doctors | 0.0269 | 0.0494 | 0.0004 | 0.001 | 0.0507 | 0.0544 | (x1,x2) | 0.0684 | 0.114 | 0.0964 | 0.1066 |
|  | x2=Materials | 0.3929 | 0.4337 | 0.0061 | 0.0085 | 0.5267 | 0.5101 | (x1,x3) | -3.5033 | 1.659 | -2.6492 | 0.6762 |
|  | x3=Outsourced services | -0.0077 | 0.0298 | -0.0001 | 0.0006 | -0.019 | 0.0804 | (x2,x3) | -51.2259 | 14.5542 | -27.4952 | 6.3442 |
| M14 | x1=Doctors | 0.0279 | 0.0082 | 0.0003 | 0.0001 | 0.0382 | 0.0084 | (x1,x2) | 0.0089 | 0.0054 | 0.1075 | 0.0409 |
|  | x2=Electricity | 3.1384 | 1.5254 | 0.0353 | 0.0277 | 0.3553 | 0.2052 | (x1,x3) | 0.3415 | 0.0397 | 0.1083 | 0.0117 |
|  | x3=Nurses | 0.0818 | 0.2078 | 0.0009 | 0.0038 | 0.3527 | 0.7162 | (x2,x3) | 38.3505 | 7.3404 | 1.0073 | 0.2865 |
| M15 | x1=Doctors | 0.0204 | 0.0459 | 0.0003 | 0.001 | 0.0418 | 0.0543 | (x1,x2) | 0.0066 | 0.017 | 0.0801 | 0.1287 |
|  | x2=Electricity | 3.0714 | 2.7041 | 0.0518 | 0.057 | 0.5215 | 0.4217 | (x1,x3) | 0.3029 | 0.3942 | 0.2291 | 0.1607 |
|  | x3=Outsourced services | 0.0673 | 0.1165 | 0.0011 | 0.0025 | 0.1825 | 0.3377 | (x2,x3) | 45.6514 | 23.2019 | 2.8579 | 1.2487 |
| M16 | x1=Doctors | 0.0087 | -0.007 | 0.0001 | -0.0001 | 0.0121 | -0.0072 | (x1,x2) | 0.0968 | -0.029 | 0.0307 | -0.0086 |
|  | x2=Nurses | 0.0899 | 0.2422 | 0.001 | 0.0045 | 0.3928 | 0.8436 | (x1,x3) | 0.0372 | -0.2017 | 0.0281 | -0.0822 |
|  | x3=Outsourced services | 0.2337 | 0.0348 | 0.0027 | 0.0006 | 0.4285 | 0.0879 | (x2,x3) | 0.3846 | 6.9578 | 0.9168 | 9.5946 |
| M17 | x1=Nurses | 0.0967 | 0.2019 | 0.0011 | 0.0039 | 0.4343 | 0.7374 | (x1,x2) | 0.7092 | -143.599 | 3.1495 | -454.272 |
|  | x2=Materials | 0.1363 | -0.0014 | 0.0016 | 0 | 0.1379 | -0.0016 | (x1,x3) | 0.0602 | 0.1433 | 2.2932 | 3.6719 |
|  | x3=Electricity | 1.6056 | 1.4089 | 0.0188 | 0.0272 | 0.1894 | 0.2008 | (x2,x3) | 0.0849 | -0.001 | 0.7281 | -0.0081 |
| M18 | x1=Nurses | 0.1141 | 0.2482 | 0.0012 | 0.0045 | 0.4707 | 0.8516 | (x1,x2) | 0.7308 | 7.7901 | 3.2454 | 24.6436 |
|  | x2=Materials | 0.1561 | 0.0319 | 0.0017 | 0.0006 | 0.145 | 0.0346 | (x1,x3) | 1.015 | 7.9408 | 2.4192 | 10.9501 |
|  | x3=Outsourced services | 0.1124 | 0.0313 | 0.0012 | 0.0006 | 0.1946 | 0.0778 | (x2,x3) | 1.3888 | 1.0193 | 0.7454 | 0.4443 |
| M19 | x1=Nurses | 0.0818 | 0.2016 | 0.0009 | 0.0038 | 0.3641 | 0.7179 | (x1,x2) | 0.0299 | 0.1276 | 1.1384 | 3.2699 |
|  | x2=Electricity | 2.7353 | 1.58 | 0.0317 | 0.0297 | 0.3198 | 0.2196 | (x1,x3) | 0.8567 | 14.5567 | 2.0419 | 20.0732 |
|  | x3=Outsourced services | 0.0955 | 0.0139 | 0.0011 | 0.0003 | 0.1783 | 0.0358 | (x2,x3) | 28.6506 | 114.0591 | 1.7936 | 6.1388 |

Calculated based on the data from Polish Association of Employers of Powiat Hospitals.
